# Optimization of proton exchange membrane fuel cell design parameters using Tianji’s horse racing optimization

**DOI:** 10.1038/s41598-026-35200-6

**Published:** 2026-01-10

**Authors:** Yacine Bouali, Khoukha Imarazene, Basem Alamri, El Madjid Berkouk

**Affiliations:** 1 Power Equipment Characterization and Diagnosis Laboratory, Department of Electrical Engineering, University of Science and Technology Houari Boumediene, P.O. Box 32, El-Alia, Algiers, 16111 Algeria; 2 Department of Electrical Engineering, College of Engineering, Taif University, P.O. Box 11099, Taif, 21944 Saudi Arabia; 3 Laboratory of Process Control (LCP), National Polytechnic School of Algiers (ENP), Algiers, Algeria

**Keywords:** Tianji’s horse racing optimization (THRO), Parameters extraction, PEMFC, Metaheuristic, Amphlett model, Energy science and technology, Engineering, Mathematics and computing

## Abstract

Accurate parameter estimation is essential for reliable modeling and performance evaluation of Proton Exchange Membrane Fuel Cells (PEMFCs). This paper introduces the application of Tianji’s Horse Racing Optimization (THRO), a recently developed metaheuristic algorithm, to precisely identify the unknown parameters of a widely used PEMFC model. The proposed THRO-based framework is evaluated on six commercial PEMFC stacks, namely NedStack PS6, Horizon 500W, BCS 500W, 250W, Avista SR-12, and Ballard Mark V, and its performance is benchmarked against five recent metaheuristic algorithms: Flood Algorithm (FLA), Educational Competition Optimizer (ECO), Kepler Optimization Algorithm (KOA), Fata Morgana Algorithm (FATA), and Spider Wasp Optimizer (SWO). Comprehensive comparative and statistical analyses demonstrate that THRO consistently achieves superior parameter identification accuracy, robustness, and solution stability across all tested PEMFC models. In particular, THRO achieves the lowest sum of squared errors (SSE), with values of 2.06, $1.12 \times 10^{-2}$, $1.16 \times 10^{-2}$, 5.25, 1.056, and 0.813 for the NedStack PS6, Horizon 500W, BCS 500W, 250W, Avista SR-12 , and Ballard Mark V PEMFC stacks, respectively. Additionally, THRO attains an extremely low standard deviation levels, indicating strong convergence reliability and resistance to premature stagnation. The obtained results confirm the effectiveness, robustness, and generalization capability of THRO for PEMFC parameter extraction, highlighting its potential as a reliable optimization tool for PEMFC parameter extraction and energy system applications.

## Introduction

The growing demand for electricity and the environmental consequences of fossil fuel consumption, such as global warming, have driven a rise in the adoption of renewable energy resources^1^. Effective energy storage techniques play a crucial role in addressing the intermittent nature of renewable energy resources, such as wind and solar power, thereby supporting the decarbonization of various sectors, including automotive, grid-connected, maritime, and residential applications^2–5^. Among the clean energy solutions that complement these systems, fuel cell devices have garnered significant attention due to their high efficiency, low emissions, and operational flexibility. In particular, Proton Exchange Membrane Fuel Cells (PEMFCs) function as efficient energy conversion technologies that electrochemically transform hydrogen into electricity, making them valuable components in renewable-energy-based power systems by enhancing overall reliability and sustainability^6–12^.

PEMFCs have emerged as a key clean energy technology in the global pursuit of carbon neutrality^6^. With rising concerns about climate change and the reliance on fossil fuels, these fuel cells efficiently convert chemical energy into electricity through a reaction between hydrogen and oxygen or another oxidizing agent, producing no harmful emissions^13^. Their adaptability makes them particularly useful in transportation, portable power, and stationary power generation^7^.

Electrochemical modeling plays a vital role in optimizing the performance of PEMFCs, serving as a foundational tool for both control system design and fault diagnosis in PEMFC stacks^14–16^. Over the past several decades, extensive research efforts have led to the development of numerous electrochemical models aimed at capturing the complex behavior of PEMFCs^15,17,18^.

These models vary in complexity and fidelity and are typically categorized into four main types: mechanistic (Physics-Based) models, which are grounded in fundamental physical and chemical principles^19^, and semi-empirical models, which combine theoretical insights with data-driven approximations to enhance computational efficiency and predictive capability^20^. Analytical models involve solving simplified mathematical equations derived from physical laws to describe the behavior of PEMFCs^21^. Empirical models are based entirely on experimental data, using statistical or curve-fitting techniques to relate input variables to output responses^22,23^.

Among the various semi-empirical models, the Amphlett model is widely recognized and accepted by the research community^24,25^. It is one of the most accurate semi-empirical models for PEMFCs, as demonstrated by its broad adoption in both static and dynamic studies. Due to its effectiveness, the Amphlett model has been extensively adopted in numerous studies, including those focusing on maximum power point prediction^26^ and dynamic system optimization^27^. Its parameter identification has become a benchmark problem in PEMFC modeling research, with studies employing advanced optimization algorithms.

In recent years, metaheuristic optimization and artificial intelligence techniques have demonstrated remarkable effectiveness across a wide range of complex engineering and intelligent computing applications. Their successful adoption extends beyond energy systems to include biomedical applications^28,29^, civil and structural engineering design^30^, power electronics^31^, antenna and electromagnetic engineering^32^, and security and intelligent protection systems^33^. Moreover, recent studies have explored human-inspired optimization frameworks, such as cultural history-based algorithms for engineering optimization^34^, comprehensive analyses and enhancements of nature-inspired optimizers^35^, and adaptive optimization strategies embedded within data-driven and deep learning models for intelligent computing tasks such as phishing detection^36^. These studies highlight the robustness, global search capability, and flexibility of metaheuristic approaches when dealing with multimodal objective functions, multi-objective optimization, and uncertain parameter spaces^37^. Such characteristics make metaheuristic algorithms particularly well suited for energy systems modeling, where complex physical interactions and nonlinear behaviors are prevalent, thereby motivating their extensive adoption in PEMFC parameter identification and optimization.

The demonstrated success of metaheuristic algorithms across these diverse domains has motivated their increasing adoption in energy-related optimization problems, particularly in the modeling and parameter identification of PEMFCs. Numerous metaheuristic algorithms have been applied to identify the nonlinear parameters of PEMFCs, offering diverse strengths in terms of convergence speed, robustness, and accuracy^38^. These algorithms include, pied kingfisher optimizer^39^, modified manta ray foraging optimization^40^, social learning-based optimization^41^, improved grey wolf optimizer^42^, enhanced salp swarm algorithm^43^, improved gorilla troops technique^44^, moth-flame optimization^45^, supply-demand-based optimization algorithm^46^, coyote optimization algorithm^47^, improved fluid search optimization algorithm^48^, walrus optimizer^49^, opposition-based arithmetic optimization algorithm^50^, and starfish optimization algorithm^51^.

In^52^, the authors introduced the Circulatory System-Based Optimization (CSBO) algorithm for estimating PEMFC parameters. The human circulatory system inspires this algorithm and, according to the reported results, it outperforms several existing approaches in terms of accuracy, speed, and stability across four different commercial stacks. The algorithm consistently produced low sum of the squared errors (SSE) and standard deviations values with efficiencies above 99%. Still, though, its real-world embedded implementation and large-scale deployment need to be demonstrated. A different approach was proposed in^53^, where an Improved Parrot Optimizer (IPO) was designed for the same parameter estimation problem. The improvement relied on opposition-based learning and a local escaping operator to enhance the search process and mitigate the algorithm’s tendency to become stuck in local optima. Validated on three PEMFC stacks, IPO achieved a noticeably smaller SSE and reduced the standard deviation by nearly 88% compared to the original optimizer. While these outcomes are encouraging, the method has only been validated in simulations, and its performance in real-world applications is still uncertain. In^54^, a hybrid strategy combining the artificial Gorilla Troops Optimizer with the Honey Badger Algorithm (GTOHBA) was presented. This combination was designed to strike a balance between global exploration and adaptive exploitation, thereby helping to mitigate issues such as premature convergence and high computational costs. Experiments on six PEMFC stacks showed that GTOHBA reached the lowest SSE, achieved minimal standard deviations, and completed runs in less than 0.4 seconds. However, its applicability to dynamic real-time conditions and high-power systems remains to be tested.

In^55^, the Artemisinin Optimization (AO) algorithm was introduced, drawing inspiration from the phases of malaria treatment. Applied to PEMFC parameter estimation, AO showed very fast convergence, low SSE values, and remarkably small standard deviations across six different stacks. It was reported to outperform nine state-of-the-art algorithms, with runtimes dropping to as little as 0.1385 seconds. However, its performance under dynamic load profiles and in large-scale PEMFC systems is still to be explored. Another contribution came from^56^, where the Parrot Optimizer (PO) was proposed. This approach, inspired by adaptive parrot behaviors, was designed for scalable parameter estimation in PEMFCs. In experiments on six commercial stacks, it surpassed nine advanced algorithms in terms of speed, accuracy, and runtime. Although PO consistently achieved low SSE values and efficient computation, further work is needed to confirm its scalability in larger or higher-dimensional systems and to assess whether it can be effectively applied to other types of fuel cells. In^57^, the Educational Competition Optimizer (ECO) was combined with a Huber loss function to estimate PEMFC parameters. The authors emphasized its ability to resist local stagnation and maintain accuracy, which was confirmed across three commercial stacks. ECO showed strong agreement with experimental polarization curves and reduced sensitivity to parameter choices. However, one potential drawback is that its computational demand may grow significantly as the system becomes more complex.

In^58^, a Cooperative Strategy-based Differential Evolution (CS-DE) algorithm was developed for PEMFC parameter estimation. By incorporating adaptive control and specialized mutation strategies, the method aimed to improve both convergence speed and robustness. Experiments on six commercial stacks showed encouraging results: CS-DE reduced SSE by up to 15% and achieved faster runtimes compared to other advanced DE variants. However, its effectiveness in more demanding settings, such as multi-objective optimization or highly dynamic PEMFC environments, has not yet been fully explored. A different study was presented in^59^, where the authors introduced an Improved Dung Beetle Optimization (IDBO) algorithm. This version combined chaotic mapping, adaptive perturbation, and osprey-inspired search strategies to boost accuracy and avoid stagnation. Tested on two commercial stacks and a self-developed 3 kW PEMFC, IDBO outperformed conventional metaheuristics in terms of SSE reduction and robustness. However, the validation so far is limited, and additional validation on larger and more dynamic systems is still required. In^60^, the War Strategy Optimization (WSO) algorithm was proposed, drawing inspiration from traditional military tactics. Applied to three benchmark stacks, WSO achieved faster convergence and lower SSE values compared to several established optimizers. The method clearly demonstrated strong search efficiency and accuracy, but whether it can scale effectively to real-time or large-scale PEMFC applications remains untested.

A Bi-Subgroup Optimization Algorithm (BSOA) was presented for PEMFC parameter estimation in^61^. To preserve diversity and reduce the likelihood of premature convergence, the strategy divides the population into two groups: one focused on exploration and the other on exploitation. BSOA outperformed both classical and more recent swarm intelligence techniques when tested on multiple benchmark stacks. However, its potential for online parameter extraction and use in large-scale, dynamic PEMFC systems remains largely untested. The Archimedes Optimization Algorithm (AOA) was used to solve the same problem in^62^. The method outperformed other algorithms in the comparison, achieving a particularly low SSE of 0.01229. Although the efficiency and accuracy reported were notable, the study emphasized that the results were limited to specific datasets and that further validation would be required, including against modern AI-based techniques and under various operating conditions. Three different versions of the Autonomous Groups Particle Swarm Optimization (AGPSO) algorithm were evaluated in^63^. The most successful of these was AGPSO3, which continuously produced the lowest total squared errors (TSE) values. Two stacks were used for validation, and AGPSO3 outperformed recent metaheuristics. It remains challenging to fine-tune group structures, and the application of this method to more complex, dynamic real-world scenarios remains untested.

For instance, the Lightning Search Algorithm (LSA) has demonstrated strong optimization capability under variable operating conditions, delivering lower error values compared to conventional techniques^64^. Similarly, the Modified Slime Mould Algorithm (MSMA), enhanced with sine-cosine mechanisms, has shown improved search efficiency and superior global performance across multiple test functions and fuel cell types^65^. Other algorithms, such as the Honey Badger Optimizer (HBO)^66^ and the Improved Crow Search Optimizer (ICSO)^67^ offer lightweight computation and dependable convergence patterns backed by validation on various commercial stacks.

Hybrid approaches such as the Particle Swarm Optimization-based Modified Golden Jackal Optimization (MGJO)^68^ integrate exploration-exploitation balances effectively, achieving the best results in some test cases. Moreover, bio-inspired algorithms like the Improved Artificial Bee Colony (IABC) benefit from evolutionary enhancements, yielding consistent performance across repeated trials^69^. Despite these advantages, each method demonstrates certain limitations. Some approaches concentrate mainly on steady-state analysis and lack dynamic analysis capabilities, whereas others can demonstrate sensitivity to fast-changing conditions or experience higher complexity in parameter tuning. These differences highlight the need for continued research in algorithmic refinement and broader applicability across diverse operational scenarios.

The No Free Lunch (NFL) theorem states that no single optimization algorithm can consistently achieve the best global solution across all types of optimization problems^70^. Consequently, researchers continue to develop and explore new metaheuristic algorithms to tackle diverse optimization challenges. Recently, a novel metaheuristic approach known as Tianji’s Horse Racing Optimization (THRO) was introduced in^71^. The novelty of THRO lies in its ability to effectively handle the complex nonlinear nature of PEMFC optimization, where many existing algorithms face difficulties with slow convergence and limited accuracy. By introducing a dynamic individual matching strategy, THRO not only accelerates convergence but also improves solution precision, making it particularly well-suited for capturing the highly nonlinear behaviors and interactions inherent in PEMFC systems. This unique capability highlights THRO’s advantage over conventional approaches and underscores its contribution to advancing reliable and efficient PEMFC optimization.

This paper presents an investigation of THRO for accurate parameter extraction of PEMFCs. The main contributions of this work are summarized as follows:

- The Tianji’s Horse Racing Optimization, a recently proposed metaheuristic algorithm^71^, is applied for the first time to PEMFC parameter estimation, demonstrating its effectiveness and robustness in a practical energy system optimization problem.
- A comprehensive comparative evaluation is performed between THRO and five recent state-of-the-art metaheuristic algorithms: Flood Algorithm (FLA)^72,73^, Educational Competition Optimizer (ECO)^74^, Kepler Optimization Algorithm (KOA)^75^, Fata Morgana Algorithm (FATA)^76^, and Spider Wasp Optimizer (SWO)^77^; to assess convergence behavior, accuracy, and robustness.
- Six widely used commercial PEMFC stacks, namely NedStack PS6, Horizon 500W, BCS 500W, 250W, Avista SR-12, and Ballard Mark V, are employed to validate the generality and scalability of the proposed optimization framework.
- The extracted parameters from the 250W PEMFC stack are further utilized for independent model validation, confirming the predictive accuracy and generalization capability of the identified model.
- The robustness of the THRO-based parameter extraction is examined under varying operating conditions, including changes in hydrogen and oxygen pressures as well as temperature variations, thereby demonstrating its reliability under realistic PEMFC operating environments.

The rest of this paper is organized as follows. The Literature Review of Metaheuristic Algorithms for PEMFC section presents a summary of metaheuristic techniques applied to PEMFC parameter extraction and discusses various stack configurations used in previous studies. The PEMFC Modeling and Problem Formulation section outlines the mathematical model of the PEMFC and defines the objective function. The Tianji’s Horse Racing Optimization section describes the THRO algorithm. The Results and Discussion section analyzes the effectiveness of the proposed approach. Finally, the Conclusion and Future Work section summarizes the key findings and outlines future research directions.

## Literature review of metaheuristic algorithms for PEMFC

A comprehensive review of recent metaheuristic and hybrid optimization algorithms applied to parameter estimation of PEMFC is presented in Table 1. The reviewed methods span a broad spectrum of recently proposed algorithmic families, including differential evolution, swarm intelligence, bio-inspired, and hybrid strategies; each method was tested across various commercial PEMFC stack models such as NedStack PS6, Ballard Mark V, and BCS 500W.

The main advantages of each method, such as robustness to nonlinearity, high estimation accuracy, fast convergence, and increased population diversity, are shown in the table. On the other hand, disadvantages and the research gap are also highlighted, including difficulties with scalability, limited validation in dynamic environments, and computational complexity.

Despite notable progress in parameter optimization, significant challenges persist, as existing studies largely emphasize algorithm development while issues of scalability, experimental validation, and efficiency under varying conditions remain unresolved. Although some approaches have demonstrated improved accuracy, convergence speed, and robustness in specific scenarios, their applicability is often restricted to particular fuel cell models or operating conditions, limiting generalizability across diverse PEMFC systems. Moreover, the integration of machine learning with traditional methods is still limited^78,79^, and little effort has been made to address multiple conflicting objectives such as accuracy, speed, and resource efficiency. These gaps underscore the need for a robust and scalable optimization strategy that leverages the strengths of different algorithms for more effective and widely applicable PEMFC parameter optimization.

**Table 1:** Review of optimization algorithms for parameter estimation of PEMFC.

Ref.	Algorithm	PEMFC types	Advantages	Disadvantages	Research gap
Evolutionary algorithms					
^80^	Differential Evolution Algorithm with Perturbation and Covariance Matrix (PCM-DE)	BCS 500W, Nedstack PS6, SR-12 W, Horizon H-12, Ballard Mark V, and STD 250W	High accuracy, fast convergence, robustness, and computational efficiency.	Performance under dynamic conditions is untested, scalability to larger PEMFC stacks is uncertain, and multi-objective optimization is not considered.	Unvalidated under dynamic PEMFC operating conditions.
Gradient-based algorithms					
^81^	Newton-Raphson-Based Optimizer (NRBO)	NedStack PS6	High accuracy, efficient search capability, and fast convergence.	Dependence on gradient information may limit performance in complex, non-smooth search spaces.	Not validated on diverse PEMFC stacks or dynamic conditions.
Swarm intelligence algorithms					
^43^	Enhanced Salp Swarm Algorithm (ESSA)	250W and BCS 500W	High accuracy, improved population diversity, fast convergence, and enhanced stability.	Complexity of the algorithm may increase computational cost, and performance under varying operating conditions (temperature, pressure) needs further validation.	Lacks validation under varying operating and dynamic conditions.
^53^	Parrot Optimizer (PO)	NedStack PS6, BCS Stack, and Ballard Mark V	Enhanced exploration capability, better avoidance of local optima, improved search efficiency, and stable convergence.	Higher computational cost due to multiple enhancements and increased complexity in implementation.	Lacks validation under real-time or dynamic PEMFC conditions.
^82^	Gazelle Optimization Algorithm (GOA)	Horizon 500W, BCS 500W, and NedStack PS6 stacks	High precision in parameter estimation, rapid convergence, and robust adaptability to polarization curve variations.	Computational complexity, potential hardware implementation challenges, and precision limitations due to fixed-point arithmetic in real-world applications.	Computational complexity and limited adaptability to real-world hardware constraints remain unresolved.
^83^	Improved Walrus Optimization Algorithm (IWOA)	Nedstack PS6, 250W, AVISTA SR-12 500W, and TEMASEK 1kW	High accuracy and fast convergence through enhanced exploration.	Increased computational complexity and limited scalability validation.	Scalability and broader applicability across larger PEMFC systems remain untested.
^84^	Enhanced Artificial Hummingbird Algorithm (EAHA)	AVISTA SR-12, BCS 500W, and 250W	High accuracy and convergence speed through hybrid foraging mechanisms and broad optimizer comparison.	Limited exploration of dynamic or real-time system behavior.	Lacks validation for transient and real-time PEMFC behavior.
^85^	Dandelion Optimization Algorithm (DOA)	250W and NedStack PS6	Fast and robust convergence with strong global search capabilities.	First-time application requires further tuning and validation for broader generalization.	Requires broader validation for generalization.
^66^	Honey Badger Optimizer (HBO)	Ballard Mark V, AVISTA SR-12, and 250W	Demonstrates strong robustness and accuracy under steady-state conditions; includes detailed sensitivity and statistical analysis; shows fast and stable convergence across multiple commercial PEMFCs.	Limited to steady-state modeling; lacks current validation under transient or real-time operational conditions.	Limited to steady-state modeling; lacks dynamic validation.
^65^	Modified Slime Mold Algorithm (MSMA)	250W, BCS 500W, AVISTA SR-12, and Temasek 1kW	Strong global search capability with enhanced local exploitation using sine-cosine mechanism; validated across benchmarks, design problems, and multiple PEMFC models; accurate and broadly applicable.	While globally effective, lacks in-depth analysis of computational cost or real-time feasibility; applicability to online or dynamic control remains to be explored.	Limits confirmation across diverse real-world PEMFC control environments.
^67^	Improved Crow Search Optimizer (ICSO)	BCS 500W and NedStack PS6	Enhanced Crow Search design leads to superior parameter estimation performance for PEMFCs, showing lower SSE than GA, Grasshopper Optimizer (GHO), and Salp Swarm Optimizer (SSO); validated on real-world PEMFCs	Focuses only on steady-state modeling; lacks testing on dynamic behavior or broader generalization beyond two specific fuel cell stacks.	Validation restricted to two stacks.
Human-based algorithms					
^60^	War Strategy Optimization (WSO)	NedStack PS6, Horizon 500W,and BCS 500W	High accuracy, efficient search capability, and fast convergence.	Performance in dynamic or high-dimensional optimization problems remains untested.	Validation is limited to benchmark test suits; broader scalability and adaptability to diverse energy system optimization tasks remain untested.
Physics-based algorithms					
^62^	Archimedes Optimization Algorithm (AOA)	250W and BCS 500W	High accuracy in parameter identification, lower SSE values compared to other optimization methods, and efficient optimization capabilities.	Dependence on real-time data, which may introduce variability, and the need for larger-scale validation under different operational conditions.	Generalizability under varying operating conditions and large-scale validation remains limited.
^64^	Lightning Search Algorithm (LSA)	Ballard Mark V, BCS 500W, and NedStack PS6	Simple and accurate algorithm with strong adaptability under different conditions; demonstrated high precision and low SSE across multiple commercial PEMFCs.	Limited discussion on robustness or scalability to more complex or real-time systems; lacks detailed comparative statistical analysis.	Validation limited to steady-state polarization fitting.
Hybrid algorithms					
^86^	Mutational Northern goshawk and Elite opposition learning-based Artificial Rabbits Optimizer (MNEARO)	BCS 500W, SR-12, Nedstack PS6, H-12, Horizon 500W and 250W	High accuracy, fast convergence, improved population diversity, and strong ability to escape local optima.	Increased computational complexity due to multiple enhancement mechanisms.	Tested only on polarization curves without transient validation.
^63^	Autonomous Groups Particle Swarm Optimization (AGPSO)	250W, and BCS 500W	Robust and statistically validated with improved accuracy and convergence stability.	Sensitive to parameter tuning and group configuration complexities.	Challenges remain in tuning group structures and ensuring scalability to real-world PEMFC systems.
^87^	Differential Evolution Ameliorated (DEA)	SR-12 500W and 250W	High parameter accuracy and fast convergence through hybrid DE–Firefly synergy.	Increased complexity from hybridization may require fine-tuning and added computational effort.	Evaluation limited to two stacks with steady-state fitting.
^88^	Hybrid Weighted Mean of Vectors and Nelder-Mead Simplex Method (INFONM)	NedStack PS6, BCS 50 W, 250W, and Ballard Mark V	Highly accurate and statistically robust with fast convergence.	Tested only on PEMFCs—broader generalizability remains unverified.	Assessment limited to four stacks without real-time validation.
^89^	Improved Adaptive Guided Differential Evolution (IAGDE)	BCS 500W, NedStack PS6, Ballard Mark V, and SR-12	Superior global and local search balance with high accuracy and fast convergence.	Increased algorithmic complexity due to multiple integrated enhancement strategies.	Scalability to larger stacks and real-time dynamic conditions remains untested.
^61^	Bi-Subgroup Optimization Algorithm (BSOA)	250W, SR-12, Ballard Mark V, BCS 500W, Temasek 1kW and NedStack PS6	Improved global convergence and population diversity by using dual subgroups and stagnation-aware updates; strong robustness for nonlinear PEMFC modeling.	Lack of theoretical speed analysis; not yet tested in real-time or online optimization scenarios.	Scalability to online parameter extraction and computational efficiency remain unaddressed.
^68^	Modified Golden Jackal Optimization (MGJO)	BCS 500W and NedStack PS6	Delivers best-in-class SSE minimization in tested PEMFC models with fast computation time; hybrid PSO approach enhances search efficiency and accuracy; outperforms multiple recent optimization algorithms.	Validation limited to static (steady-state) models; lacks dynamic/transient behavior modeling and fuel cell degradation analysis.	Robustness under aging effects and long-term dynamic operation remains unverified.
^69^	Improved Artificial Bee Colony (IABC)	250W, BCS 500W and NedStack PS6	Hybridized with genetic and differential evolution strategies for enhanced convergence speed and robustness; delivers precise parameter estimates across multiple PEMFC models with minimal SSE and strong statistical performance (low STD, validated via ANOVA).	Evaluation limited to steady-state conditions; lacks testing under dynamic or rapidly changing operating scenarios, which is essential for real-world PEMFC applications.	Accuracy under rapidly changing operating conditions remains unverified.

This study employs a combination of well-established algorithms and several recently introduced metaheuristic approaches that, to the best of our knowledge, have not yet been applied to PEMFC parameter estimation in the existing literature. The comparative algorithms were selected primarily based on their novelty and relevance, as all selected methods were proposed after 2023. This selection enables a fair and up-to-date evaluation of the proposed THRO algorithm against recent optimization strategies. To maintain conciseness and avoid excessive length, brief descriptions of each algorithm are presented in Table 2, rather than providing detailed explanations in the main text.

**Table 2:** A summary of the metaheuristic algorithms used in this paper.

Optimization algorithm	Summary
THRO	See the THRO section below.
FLA	Flood Algorithm (FLA): Nature-inspired meta-heuristic based on water movement during floods^72,73^. Simulates slope-driven flow, soil permeability, and fluctuating water levels. Two phases: (1) Regular movement guiding solutions toward optima, (2) Flooding phase introducing randomness. Weak solutions replaced periodically.
ECO	Educational Competition Optimizer (ECO): Inspired by educational resource competition^74^. Simulates student progression through elementary, middle, and high school phases. Transitions from exploration to exploitation. Efficiently narrows solution pool with strong benchmark performance.
KOA	Kepler Optimization Algorithm (KOA): Physics-inspired approach using Kepler’s planetary motion laws^75^. Models solutions as planets orbiting best solution (Sun). Updates positions via orbital dynamics. Balances exploration-exploitation.
FATA	Fata Morgana Algorithm (FATA): Swarm intelligence inspired by mirages^76^. Uses Mirage Light Filtering (MLF) for exploration and Light Propagation Strategy (LPS) for exploitation. Balances population/individual search abilities.
SWO	Spider Wasp Optimization (SWO): Nature-inspired algorithm based on hunting/nesting behaviors of spider wasps^77^. Employs diverse update strategies. Effective for benchmarks and engineering problems.

## PEMFC modeling and problem formulation

### PEMFC modeling

**Fig. 1:**
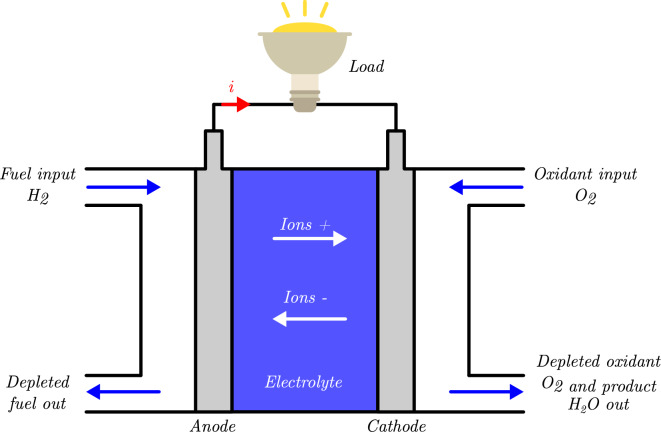
The schematic diagram of a PEMFC.

The proton exchange membrane fuel cell, also referred to as the polymer electrolyte membrane fuel cell, is an electrochemical device that efficiently converts chemical energy directly into electrical energy. Figure 1 illustrates the fundamental structure and operating principle of the PEMFC. At the anode, hydrogen (${\hbox {H}}_{2}$) undergoes catalytic dissociation, where electrons are separated from protons within the catalyst layers. The protons permeate through the proton-conducting membrane to reach the cathode while the electrons traverse an external circuit, generating electrical power. Simultaneously, at the cathode, oxygen (${\hbox {O}}_{2}$) reacts with the incoming protons and electrons to form water (${\hbox {H}}_{2}{\hbox {O}}$) as the primary byproduct.

The chemical reactions at the anode, cathode, and the overall reaction of this fuel cell are given as:

Anode: $$2{\hbox {H}}_{2}$$ $$\longrightarrow$$ $$4{\hbox {e}}^-$$ + $$4{\hbox {H}}^+$$

Cathode: $$4{\hbox {e}}^-$$ + $$4{\hbox {H}}^+$$ + $${\hbox {O}}_{2}$$ $$\longrightarrow$$ $$2{\hbox {H}}_{2}\hbox {O}$$

Net: $$2{\hbox {H}}_{2}$$ + $${\hbox {O}}_{2}$$ $$\longrightarrow$$ $$2{\hbox {H}}_{2}\hbox {O}$$

**Fig. 2:**
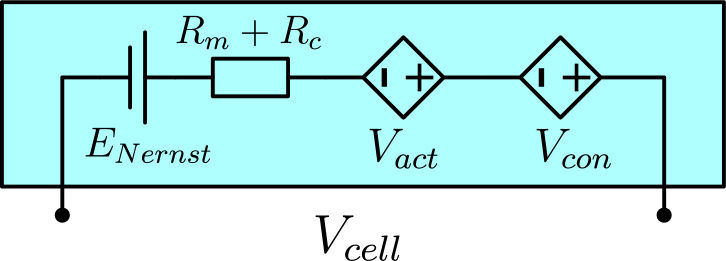
Electrical model of a PEMFC.

In practical PMEFC systems, usually $N_{cell}$ single PEMFC are stacked together. Based on the electrical model of a PEMFC in Fig. 2, the terminal voltage *V* generated by the PEMFC stack can be expressed as:

1 $$\begin{aligned} V=N_{cell} V_{cell} \end{aligned}$$

The cell voltage $V_{cell}$ expressed as follow:

2 $$\begin{aligned} V_{cell} = E_{Nernst} - V_{act} - V_{ohm} - V_{con} \end{aligned}$$

The reversible voltage, $E_{Nernst}$, represents the thermodynamic equilibrium potential in the absence of current flow (i.e., the open circuit voltage). This potential can be determined using a modified form of the Nernst equation, as expressed in (3)^46,53^. The modified Nernst equation is calculated under standard conditions, specifically a temperature of 298.15 K and a pressure of 1 bar, ensuring accurate computation of $E_{Nernst}$ under these baseline parameters.

3 $$\begin{aligned} \begin{aligned} E_{Nernst}&= 1.229 - 0.85 \times 10^{-3} (T - 298.15) \\&+ 430.85 \times 10^{-7} T \left[ Ln(P_{H_2}\sqrt{P_{O_2}})\right] \end{aligned} \end{aligned}$$

The cell temperature is denoted by *T*, with the partial pressures of oxygen and hydrogen represented as $P_{H_2}$ and $P_{O_2}$ as in (4) and (5), respectively. If the reactants are air and ${\hbox {H}}_{2}$, then $P_{O_2}$ should be calculated using (6)^46^.

4 $$\begin{aligned} & P_{H_2} = 0.5 R_{HA} P_{H_{2}O} \left[ \left( exp(\frac{1.635}{T^{1.334}} \frac{I}{A}) \frac{R_{HA}P_{H_{2}O}}{P_A}\right) ^{-1}-1\right] \end{aligned}$$

5 $$\begin{aligned} & P_{O_2} = R_{HC} P_{H_{2}O} \left[ \left( exp(\frac{4.192}{T^{1.334}} \frac{I}{A}) \frac{R_{HC}P_{H_{2}O}}{P_C}\right) ^{-1}-1\right] \end{aligned}$$

here, $R_{HA}$ and $R_{HC}$ are the relative humidity of vapour in the anode and the cathode, respectively. $P_{H_{2}O}$ is the saturation pressure of water, which can be expressed (7), $P_A$ is the anode pressure at the inlet, while that at the cathode is $P_C$, the area of the cell is *A*, while the current is *I*.

6 $$\begin{aligned} & P_{O_2} = \frac{P_C - R_{HC} P_{H_{2}O}}{(1+\frac{0.79}{0.21})exp(\frac{0.291}{T^{0.832}}\frac{I}{A})} \end{aligned}$$

7 $$\begin{aligned} & \begin{aligned} \log _{10}(P_{H_{2}O})&= 29.5 \times 10^{-3} (\Delta T) -91.8 \times 10^{-6} (\Delta T)^2 \\ &+14.4 \times 10^{-8} (\Delta T)^3 - 2.18 \end{aligned} \end{aligned}$$

where, $$\Delta T = T - 273.15$$

The concentration voltage drop is represented by $V_{act}$, which is caused by the slowness of the reactions taking place on the active surface of the anode and cathode. A semi-empirical equation can be used to compute $V_{act}$ as expressed in Eq. (8)^53^.

8 $$\begin{aligned} V_{act} = - \left[ \xi _1 + \xi _2 T +\xi _3 T Ln(C_{O_2}) +\xi _4 T Ln(I)\right] \end{aligned}$$

The semi-empirical coefficients $\xi _1$ to $\xi _4$ in the Butler-Volmer equation have well-defined physical interpretations based on kinetic, thermodynamic, and electrochemical principles. The concentration of dissolved oxygen at the cathode’s catalytic interface ($C_{O_2}$) can be determined using Henry’s Law, as expressed in (9)^53^.

9 $$\begin{aligned} C_{O_2} = \frac{P_{O_2}}{5.08 \times 10^{6}} exp\left( \frac{498}{T}\right) \end{aligned}$$

The ohmic voltage drop caused by resistance, $V_{ohm}$ in (10) is influenced by three factors: the equivalent membrane resistance $R_m$, the contact resistance for proton conduction $R_c$, and the stack current *I*^46,53^.

10 $$\begin{aligned} V_{ohm} = I (R_m + R_c) \end{aligned}$$

The ionic resistance, $R_c$, is treated as a constant due to the relatively narrow operating temperature range of the PEMFC. The electronic resistance, $R_m$, is defined in (11).

11 $$\begin{aligned} R_m = \frac{\rho _m l}{A} \end{aligned}$$

here, *l* represents the thickness of the membrane, and $\rho _m$, is the specific resistivity for the flow of hydrated protons, which can be expressed using an empirical equation in (12)^46^.

12 $$\begin{aligned} \rho _m = \frac{181.6 \left( 1 + 0.03 \frac{I}{A} + 0.062 \left( \frac{T}{303} \right) ^2 \left( \frac{I}{A} \right) ^{2.5} \right) }{\left( \lambda - 0.634 - 3 \frac{I}{A} \right) \exp \left( 4.18 \frac{T - 303}{T} \right) } \end{aligned}$$

where, $\lambda$ is a semi empirical parametric coefficient that represents the water content of the membrane. It is one of the unknown parameters that need to be determined.

The concentration voltage loss, $V_{con}$, is given by (13)^46^, $V_{con}$ results from the pressure drop and the decrease in reactant concentration due to fluid resistance during cell operation. This loss leads to a reduction in output voltage and is influenced by the system’s supply and the electrical current from the cell.

13 $$\begin{aligned} V_{con} = -\beta \ln \left( 1 - \frac{J}{J_{max}}\right) \end{aligned}$$

here, $J_{max}$ is the limiting current density, which corresponds to the maximum supply speed, and $\beta$ is a parametric coefficient that depends on the cell type and its operational state. Meanwhile, *J* represents the actual current density.

By combining the equations from (1) to (13), seven parameters, $X = \left[ \xi _1, \xi _2, \xi _3, \xi _4, \lambda ,\right.$ $\left. \beta , R_c\right]$, are unknown and must be determined using optimization algorithms.

### Objective function

In the model described from Eqs. (1) to (13), the operational parameters $T, R_{HA},$ $R_{HC},$ $P_{A}, P_{C}, P_{H_2}, P_{O_2}$ are measured and vary depending on the operating conditions. Conversely, the physical parameters $\xi _1, \xi _2, \xi _3, \xi _4, \lambda , \beta , R_c$ must be determined based on the experimental voltage-current (V–I) data from the PEMFC stack. To achieve this, an appropriate objective function is defined and minimized as given in (14). This objective function is formulated as the sum of squared errors (SSEs), representing the discrepancy between the experimental data and the model predictions.

14 $$\begin{aligned} SSE= \sum _{d=1}^{D} \sum _{i=1}^{N}\left[ V_{mes}(i,d) - V_{est}(i,d)\right] ^2 \end{aligned}$$

where, *D* represent the number of datasets, $V_{mes}$ represents the measured voltage of the PEMFC stack, $V_{est}$ denotes the estimated voltage obtained from the PEMFC stack mathematical model, and *N* is the total number of experimental V–I data points in each dataset.

The seven unknown parameters of the PEMFC stack model are constrained within predefined lower and upper boundaries, as outlined in (15). The specific range for each parameter utilized in this study is provided in Table 3.

15 $$\begin{aligned} \begin{aligned} \xi _{i}^{min}&\le \xi _i \le \xi _{i}^{max} \\ \lambda ^{min}&\le \lambda \le \lambda ^{max} \\ \beta ^{min}&\le \beta \le \beta ^{max} \\ R_{c}^{min}&\le R_c \le R_{c}^{max} \end{aligned} \end{aligned}$$

**Table 3:** Lower and upper bounds on PEMFC model parameters.

Parameter	Min	Max
$\xi _1$	$-1.19969$	$-0.8532$
$\xi _2 \times 10^{-3}$	1	5
$\xi _3 \times 10^{-5}$	3.6	9.8
$\xi _4 \times 10^{-5}$	$-26$	$-9.54$
$\lambda$	10	24
$\beta ~(\textrm{V})$	0.0136	0.5
$R_c \times 10^{-4}~(\Omega )$	1	8

To assess the performance and robustness of the optimization algorithm in estimating PEMFC parameters, three statistical metrics were employed: Mean Absolute Error (MAE), Relative Error (RE), and Root Mean Square Error (RMSE). These metrics are computed based on the SSE obtained from $N_{Run}$ independent runs. MAE and RMSE quantify the average and squared deviations from the minimum SSE, respectively, while RE evaluates the overall deviation relative to the best-performing solution. Together, these indicators provide a comprehensive evaluation of both accuracy and reliability. Their corresponding mathematical expressions are given as follows:

16 $$\begin{aligned} & \text {MAE} = \frac{1}{N_{Run}} \sum _{i=1}^{N_{Run}} (\text {SSE}_i - \text {SSE}_{\min }) \end{aligned}$$

17 $$\begin{aligned} & \text {RE} = \frac{\sum _{i=1}^{N_{Run}} (\text {SSE}_i - \text {SSE}_{\min })}{\text {SSE}_{\min }} \end{aligned}$$

18 $$\begin{aligned} & \text {RMSE} = \sqrt{\frac{1}{N_{Run}} \sum _{i=1}^{N_{Run}} (\text {SSE}_i - \text {SSE}_{\min })^2} \end{aligned}$$

## Tianji’s horse racing optimization

The Tianji’s Horse Racing Optimization (THRO) algorithm draws inspiration from the ancient Chinese story of Tianji’s horse racing, a well-known example of strategic thinking from over 2000 years ago^71^. In the story, Tianji, a military leader, consistently lost races against the king until his advisor Sunbin proposed a clever strategy: sacrifice the first round by pitting his weakest horse against the king’s strongest, then win the next two rounds by matching his strongest horse against the king’s medium and his medium against the king’s weakest. This smart allocation of resources inspired the THRO algorithm’s design, where different levels of search capability (exploration and exploitation) are balanced by strategically assigning weaker or stronger agents in a way that mirrors Tianji’s adaptive and iterative approach to winning.

### Competition

In the THRO algorithm, there are two populations; Tianji’s horses and the King’s horses, each consisting of *n* horses, where Tianji’s horses can be represented as:

19 $$\begin{aligned} X_T=\begin{bmatrix} x_{T1} \\ \dots \\ x_{Ti} \\ \dots \\ x_{Tn} \end{bmatrix}=\begin{bmatrix} x_{T1}^{1} & \dots & x_{T1}^{j} & \dots & x_{T1}^{d}\\ \dots & & \dots & & \dots \\ x_{Ti}^{1} & \dots & x_{Ti}^{j} & \dots & x_{Ti}^{d}\\ \dots & & \dots & & \dots \\ x_{Tn}^{1} & \dots & x_{Tn}^{j} & \dots & x_{Tn}^{d} \end{bmatrix} \end{aligned}$$

Let $x_{Ti}$ denote the *i*-th horse in Tianji’s population. Each horse is characterized by multiple attributes, such as breed, physique, age, etc; which collectively influence its running speed. The term $x_{Ti}^{j}$ represents the *j*-th attribute of the *i*-th horse in Tianji’s group, where *d* is the total number of attributes. In the context of a minimization problem, a horse’s fitness value corresponds to its running speed; a lower fitness value indicates a faster horse.

Similarly, the King’s horses can be represented as:

20 $$\begin{aligned} X_K=\begin{bmatrix} x_{K1} \\ \dots \\ x_{Ki} \\ \dots \\ x_{Kn} \end{bmatrix}=\begin{bmatrix} x_{K1}^{1} & \dots & x_{K1}^{j} & \dots & x_{K1}^{d}\\ \dots & & \dots & & \dots \\ x_{Ki}^{1} & \dots & x_{Ki}^{j} & \dots & x_{Ki}^{d}\\ \dots & & \dots & & \dots \\ x_{Kn}^{1} & \dots & x_{Kn}^{j} & \dots & x_{Kn}^{d} \end{bmatrix} \end{aligned}$$

here $x_{Ki}$ is the *i*-th horse in King’s horses.

In the original story, Tianji and the King each race with three horses. The THRO algorithm generalizes this to *n* horses per side ($n > 3$), with each side’s horses sorted in descending order of speed. Horses are categorized into *n* classes, from fastest (first-class) to slowest (*n*th-class). In each iteration, *n* races occur between corresponding horses, which are then removed from the current population. The THRO algorithm employs five competition strategies as follow:

#### Scenario 1

If Tianji’s slowest horse is faster than the King’s slowest, they are matched, and Tianji wins. To maintain this advantage, the algorithm updates Tianji’s slowest horse based on the influence of Tianji’s fastest horse and the overall quality gap between the two populations. The update rule is:

21 $$\begin{aligned} \left\{ \begin{aligned} v_{T_{si}}(t+1) = {}&\Bigl ( p \cdot x_{T_{si}}(t) + (1-p) \cdot x_{T_{f}}(t) \\&+ R \cdot \bigl ( x_{T_{f}}(t) - x_{T_{si}}(t) \\&+ p \cdot (\bar{x}_T(t) - \bar{x}_K(t)) \bigr ) \Bigr ) \cdot \alpha + \beta \\ T_{si} = {}&T_{si} - 1 \end{aligned} \right. \end{aligned}$$

22 $$\begin{aligned} \alpha = 1 + round(0.5 \times (0.5 +rand)) \times n_1 \end{aligned}$$

23 $$\begin{aligned} \beta = round(0.5 \times (0.1 +rand)) \times n_2 \end{aligned}$$

24 $$\begin{aligned} p=1-\frac{t}{T} \end{aligned}$$

25 $$\begin{aligned} R=L\times B \end{aligned}$$

26 $$\begin{aligned} L=\frac{u.\sigma }{|v |^{1/b}} \end{aligned}$$

27 $$\begin{aligned} \sigma = \left( \frac{\Gamma (1+b) \times sin (\frac{\pi b}{2})}{\Gamma (\frac{1+b}{2}) \times b \times 2^{\frac{b-1}{2}}}\right) ^{1/b} \end{aligned}$$

28 $$\begin{aligned} B= \left[ b_1, \dots , b_k, \dots , b_d\right] \end{aligned}$$

29 $$\begin{aligned} b(k){\left\{ \begin{array}{ll} 1 \qquad if \quad k == g(l)\\ 0 \qquad else \end{array}\right. } \end{aligned}$$

30 $$\begin{aligned} g=randperm(d) \end{aligned}$$

31 $$\begin{aligned} l = 1, \dots , \Big \lceil \sin \left( \frac{\pi r_1}{2} \right) \times d \Big \rceil \end{aligned}$$

here, $x_{T_{si}}$ denotes Tianji’s current slowest horse, and $T_{si}$ is the number of Tianji’s current slowest horse. The variables $n_1, n_2, u,$ and *v* follow a standard normal distribution. $x_{T_{f}}$ is Tianji’s fastest horse, while $\bar{x}_T$ and $\bar{x}_K$ represent the average quality of Tianji’s and the King’s horse populations, respectively. The parameter *p* is a weighting factor, $\Gamma$ is the standard Gamma function with $b = 1.5$, and *R* is the running factor that enables the algorithm to perform a Levy flight-based search in randomly selected dimensions of the solution space. $\beta$ is a mutation factor introduced to increase population diversity and promote a more efficient search process.

To catch up, the King’s slowest horse $x_{K_{si}}$ is updated based on Tianji’s slowest horse $x_{T_{si}}$. The update rule is:

32 $$\begin{aligned} \left\{ \begin{aligned} v_{K_{si}}(t+1) = {}&\Bigl ( p \cdot x_{K_{si}}(t) + (1-p) \cdot x_{T_{si}}(t) \\&+ R \cdot \bigl ( x_{T_{si}}(t) - x_{K_{si}}(t) \\&+ p \cdot (\bar{x}_T(t) - \bar{x}_K(t)) \bigr ) \Bigr ) \cdot \alpha + \beta \\ K_{si} = {}&K_{si} - 1 \end{aligned} \right. \end{aligned}$$

here, $x_{K_{si}}$ is updated to approach $x_{T_{si}}$, while accounting for the overall strength difference between the two populations and the level of $x_{T_{si}}$. And $K_{si}$ is the number of King’s current slowest horse.

#### Scenario 2

If Tianji’s slowest horse is slower than the King’s slowest, it is matched against the King’s fastest horse. Although Tianji loses the round, this strategy sacrifices the weakest horse to counter the King’s strongest. Knowing that the slowest horse cannot outperform any of the King’s, the algorithm updates Tianji’s slowest horse based on a randomly selected horse from Tianji’s population. The update rule is:

33 $$\begin{aligned} \left\{ \begin{aligned} v_{T_{si}}(t+1) = {}&\Bigl ( p \cdot x_{T_{si}}(t) + (1-p) \cdot x_{T_{r1}}(t) \\&+ R \cdot \bigl ( x_{T_{r1}}(t) - x_{T_{si}}(t) \\&+ p \cdot (\bar{x}_T(t) - \bar{x}_K(t)) \bigr ) \Bigr ) \cdot \alpha + \beta \\ T_{si} = {}&T_{si} - 1 \end{aligned} \right. \end{aligned}$$

with $x_{T_{r1}}$ is a randomly chosen horse from Tianji’s horse population.

To maintain his lead, the King aims to make his horses outperform Tianji’s. Thus, the algorithm updates the King’s fastest horse based on the best horse in his population. The update rule is:

34 $$\begin{aligned} \left\{ \begin{aligned} v_{K_{fi}}(t+1) = {}&\Bigl ( p \cdot x_{K_{fi}}(t) + (1-p) \cdot x_{K_{f}}(t) \\&+ R \cdot \bigl ( x_{K_{f}}(t) - x_{K_{fi}}(t) \\&+ p \cdot (\bar{x}_T(t) - \bar{x}_K(t)) \bigr ) \Bigr ) \cdot \alpha + \beta \\ K_{fi} = {}&K_{fi} + 1 \end{aligned} \right. \end{aligned}$$

here, $x_{K_{f}}$ denotes the fastest horse in the King’s population, and $K_{fi}$ is the number of King’s current fastest horse.

#### Scenario 3

If Tianji’s fastest horse is quicker than the King’s, and their slowest horses are evenly matched, Tianji’s fastest horse races the King’s and wins. To preserve this advantage, the algorithm updates Tianji’s fastest horse based on the best in Tianji’s population. The update rule is:

35 $$\begin{aligned} \left\{ \begin{aligned} v_{T_{fi}}(t+1) = {}&\Bigl ( p \cdot x_{T_{fi}}(t) + (1-p) \cdot x_{T_{f}}(t) \\&+ R \cdot \bigl ( x_{T_{f}}(t) - x_{T_{fi}}(t) \\&+ p \cdot (\bar{x}_T(t) - \bar{x}_K(t)) \bigr ) \Bigr ) \cdot \alpha + \beta \\ T_{fi} = {}&T_{fi} + 1 \end{aligned} \right. \end{aligned}$$

here, $T_{fi}$ is the number of Tianji’s current fastest horse, and $x_{T_{f}}$ is the fastest horse in Tianji’s population.

To catch up, the King’s current fastest horse is updated based on Tianji’s current fastest horse. The update rule is:

36 $$\begin{aligned} \left\{ \begin{aligned} v_{K_{fi}}(t+1) = {}&\Bigl ( p \cdot x_{K_{fi}}(t) + (1-p) \cdot x_{T_{fi}}(t) \\&+ R \cdot \bigl ( x_{T_{fi}}(t) - x_{K_{fi}}(t) \\&+ p \cdot (\bar{x}_T(t) - \bar{x}_K(t)) \bigr ) \Bigr ) \cdot \alpha + \beta \\ K_{fi} = {}&K_{fi} + 1 \end{aligned} \right. \end{aligned}$$

#### Scenario 4

If Tianji’s slowest horse matches the King’s, but his fastest is slower than the King’s fastest, Tianji sacrifices his slowest horse against the King’s strongest and loses. Since the loss is inevitable, the algorithm updates Tianji’s slowest horse using a randomly selected horse from Tianji’s population. The update rule is:

37 $$\begin{aligned} \left\{ \begin{aligned} v_{T_{si}}(t+1) = {}&\Bigl ( p \cdot x_{T_{si}}(t) + (1-p) \cdot x_{T_{r2}}(t) \\&+ R \cdot \bigl ( x_{T_{r2}}(t) - x_{T_{si}}(t) \\&+ p \cdot (\bar{x}_T(t) - \bar{x}_K(t)) \bigr ) \Bigr ) \cdot \alpha + \beta \\ T_{si} = {}&T_{si} - 1 \end{aligned} \right. \end{aligned}$$

here, $x_{T_{r2}}$ is a randomly selected horse from Tianji’s population.

For the King, as in Scenario 2, the algorithm updates his fastest horse based on the best horse in his population. The update rule is:

38 $$\begin{aligned} \left\{ \begin{aligned} v_{K_{fi}}(t+1) = {}&\Bigl ( p \cdot x_{K_{fi}}(t) + (1-p) \cdot x_{K_{f}}(t) \\&+ R \cdot \bigl ( x_{K_{f}}(t) - x_{K_{fi}}(t) \\&+ p \cdot (\bar{x}_T(t) - \bar{x}_K(t)) \bigr ) \Bigr ) \cdot \alpha + \beta \\ K_{fi} = {}&K_{fi} + 1 \end{aligned} \right. \end{aligned}$$

#### Scenario 5

If both the fastest and slowest horses of Tianji and the King run at equal speeds, Tianji sacrifices his slowest horse against the King’s strongest and loses. The algorithm applies the same update strategy as in Scenario 4. Tianji’s slowest horse is updated as:

39 $$\begin{aligned} \left\{ \begin{aligned} v_{T_{si}}(t+1) = {}&\Bigl ( p \cdot x_{T_{si}}(t) + (1-p) \cdot x_{T_{r3}}(t) \\&+ R \cdot \bigl ( x_{T_{r3}}(t) - x_{T_{si}}(t) \\&+ p \cdot (\bar{x}_T(t) - \bar{x}_K(t)) \bigr ) \Bigr ) \cdot \alpha + \beta \\ T_{si} = {}&T_{si} - 1 \end{aligned} \right. \end{aligned}$$

here, $x_{T_{r3}}$ is a randomly selected horse from Tianji’s population. The King’s current fastest horse is then updated as:

40 $$\begin{aligned} \left\{ \begin{aligned} v_{K_{fi}}(t+1) = {}&\Bigl ( p \cdot x_{K_{fi}}(t) + (1-p) \cdot x_{K_{f}}(t) \\&+ R \cdot \bigl ( x_{K_{f}}(t) - x_{K_{fi}}(t) \\&+ p \cdot (\bar{x}_T(t) - \bar{x}_K(t)) \bigr ) \Bigr ) \cdot \alpha + \beta \\ K_{fi} = {}&K_{fi} - 1 \end{aligned} \right. \end{aligned}$$

### Training

Since THRO is an iterative search algorithm, solutions from the previous iteration must be effectively reinforced in the next. To prevent stagnation, a training strategy is introduced. After each race, both populations undergo training to boost competitiveness. Horses train with others of varying speeds to gradually improve, and with the fastest horse to reach their full potential. This strategy is mathematically expressed as:

41 $$\begin{aligned} v_{T_{i}}^{j}(t+1)= {\left\{ \begin{array}{ll} x_{T_{i}}^{j}(t) + L_T \times (x_{T_{r4}}^{j}- x_{T_{r5}}^{j}) \quad \text { if } rand < 0.5\\ x_{T_{f}}^{j}(t) + M_T \times (x_{T_{f}}^{j} (t)- x_{T_{i}}^{j}(t) ) \quad \text {else} \end{array}\right. } \end{aligned}$$

42 $$\begin{aligned} {\left\{ \begin{array}{ll} L_T = 0.2 \times L\\ M_T = \frac{1}{2} \times \left( 1 + \frac{1}{1000} \times (1- \frac{t}{T})^2 \times sin(\pi \times rand)\right) \end{array}\right. } \end{aligned}$$

43 $$\begin{aligned} v_{K_{i}}^{j}(t+1)= {\left\{ \begin{array}{ll} x_{K_{i}}^{j}(t) + L_K \times (x_{K_{r1}}^{j}- x_{K_{r2}}^{j}) \quad \text { if } rand < 0.5\\ x_{K_{f}}^{j}(t) + M_K \times (x_{K_{f}}^{j} (t)- x_{K_{i}}^{j}(t) ) \quad \text {else} \end{array}\right. } \end{aligned}$$

44 $$\begin{aligned} {\left\{ \begin{array}{ll} L_K = 0.2 \times L\\ M_K = \frac{1}{2} \times \left( 1 + \frac{1}{1000} \times (1- \frac{t}{T})^2 \times sin(\pi \times rand)\right) \end{array}\right. } \end{aligned}$$

here, $x_{T_{i}}^{j}$ is the *j*-th attribute of the $T_i$-th horse in Tianji’s population, and $x_{K_{i}}^{j}$ is the *j*-th attribute of the $K_i$-th horse in the King’s population. $x_{T_{f}}^{j}$ and $x_{K_{f}}^{j}$ represent the *j*-th attribute of the fastest horse in Tianji’s and the King’s populations, respectively. $T_{r4}$ and $T_{r5}$ are the number of two randomly selected horses from Tianji’s population, while $K_{r1}$ and $K_{r2}$ are the number of two randomly selected horses from the King’s population. *T* denotes the maximum number of iterations. $L_T$ and $M_T$ are training factors for Tianji’s population, and $L_K$ and $M_K$ are training factors for the King’s population.

### Procedure of THRO

**Fig. 3:**
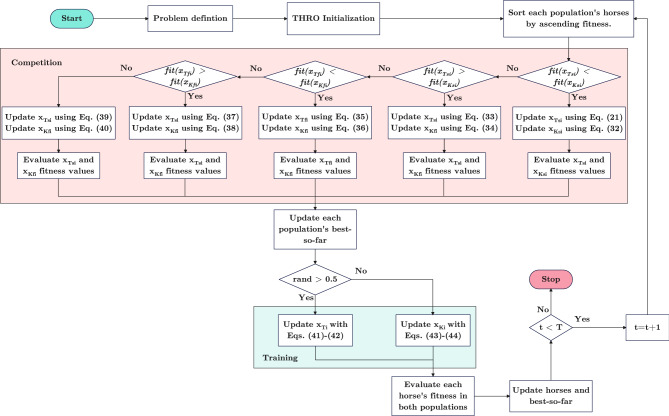
The flowchart of THRO.

The THRO algorithm begins by initializing control parameters, including the population sizes for Tianji and the King, and the maximum number of iterations. Both horse populations are randomly generated within the search space. In each iteration, the horses are regrouped and sorted in descending order of speed. Then, for each of the *n*-rounds, specific update strategies are applied based on five competition scenarios: Scenario 1 updates Tianji’s slowest horse using his fastest, and updates the King’s slowest using Tianji’s slowest; Scenario 2 updates Tianji’s slowest with a random Tianji horse, and the King’s fastest with his own fastest; Scenario 3 updates both fastest horses using Tianji’s fastest; Scenarios 4 and 5 update Tianji’s slowest with a random Tianji horse and the King’s fastest with his own fastest. After all races, candidate solutions are generated, and each horse is updated if the candidate performs better. A training phase follows, where horses improve through interaction with others and the population’s best. This loop continues until the stopping criterion is met, after which the best horse found is returned. The flowchart of THRO, shown in Fig. 3, captures this process.

The evaluation of computational complexity plays a crucial role in assessing the performance of new optimization algorithms. In the case of the THRO, several key parameters directly influence its computational cost. These include the population size of Tianji’s and King’s horses (*n*), the variables dimension of the problem (*d*), and the maximum number of iterations (*T*). Taking these factors into account, the overall computational complexity of THRO can be expressed as follows^71^.

$$\begin{aligned} O(THRO) \simeq O(Tn(d+log(n))) \end{aligned}$$

## Results and discussion

This section applies the proposed THRO algorithm alongside five recently introduced metaheuristic algorithms to accurately identify the seven unknown parameters of six widely studied PEMFC stacks: NedStack PS6, Horizon 500W, BCS 500W, 250W, Avista SR-12, and Ballard Mark V^53,59,85–87,91,92^. These stacks were selected due to their frequent use in prior research, The datasets of these stacks are available in their corresponding references as follows: NedStackPS6^53,85^, Horizon-500W^46^, BCS500W^46,53,92^, 250W^46^, Avista SR-12^87^, and Ballard Mark V^53^. The characteristics and operating conditions of the PEMFC stacks are drawn from reference^46,53,59,85–87,91–93^ and are summarized in Table 4.

**Table 4:** The specifications and operating conditions of PEMFC stacks.

PEMFC stacks	$N_{cell}$	$A(cm^2)$	$l(\mu m)$	$J_{max}(A/cm^2)$	*T*(*K*)	$P_A(bar)$	$P_C(bar)$	$R_{HA}$	$R_{HC}$	$P_{H_{2}O}(atm)$	$P_{O_2}(atm)$
NedStack PS6^53^	65	240	178	5	343	–	–	–	–	1	1
Horizon 500W^46,94^	36	52	25	0.51923	$\le$338.15	–	–	-	–	0.55	1
BCS 500W^46,93^	32	64	178	0.469	333	–	–	–	–	1	0.2075
250W^46,85^	24	27	127	0.860	343.15–353.15	1.0–3.0	1.0–5.0	1	1	–	–
Avista SR-12^87,92^	48	62.5	25	0.672	323	–	–	–	–	1.47628	0.2095
Ballard Mark V^53^	35	50.6	178	1.50	343	-	–	–	–	1	1

These stacks were selected because their experimental V–I data are openly available and reproducible from the literature, allowing readers to verify and replicate the results^46,53,87^. For the 250W stack, four different datasets are available^46^. The first two datasets (3/5 bar at 353.15 K and 1/1 bar at 343.15 K) are used for parameter extraction, while the remaining two datasets (2.5/3 bar at 343.15 K and 1.5/1.5 bar at 343.15 K) are used for model validation.

To ensure a fair comparison among the algorithms, the population size and the maximum number of iterations for all methods are uniformly set to 50 and 1000, respectively. All simulations in this study are conducted using MATLAB R2021a on a personal computer equipped with an Intel Core i5-3230M CPU at 2.60 GHz and 8.00 GB of RAM. The control parameters of the comparative algorithms are summarized in Table 5. These parameters were initially selected based on established practices reported in the literature and the original publications of each algorithm.

**Table 5:** The control parameters of the different metaheuristic algorithms.

Algorithms	Control parameters
THRO	No parameters
FLA	Number of weak population that will be replaced, $N_e=5$
ECO	Learning habit boundary, $H = 0.5$
	Proportion of primary school (Stage 1), $G_1=0.2$
	Proportion of middle school (Stage 2), $G_2=0.1$
KOA	Initial value, $\mu _o=0.1$
	The constant, $\gamma =15$
	The constant, $\overline{T} =15$
FATA	The reflectance of the reflection strategy, $\alpha =0.2$
SWO	Hunt–mate trade-off, $T_R=0.3$
	The Crossover probability, $C_r=0.2$
	Representing the minimum population size, $N_{min}=20$

Table 6 presents the statistical comparison of the final SSE values obtained from various fuel cell models under different optimization algorithms. Given the stochastic nature of these algorithms, their results can vary across executions. This variability arises from the intentional incorporation of randomness in their design, which facilitates exploration of the search space and helps avoid local optima. To ensure a robust and reliable evaluation, each algorithm was executed independently 30 times in this study.

The statistical analysis across six PEMFC stacks demonstrates the superior performance of the THRO algorithm in terms of accuracy, consistency, and reliability. As shown in Table 6, THRO achieves a lower minimum of SSE values for each PEMFC stack accompanied by near-zero standard deviations (std), on the order of $10^{-12}$ to $10^{-16}$. This behavior signifies convergence to the same optimal solution in each run, an outcome that is particularly evident in the NedStack PS6 case, where THRO attains an SSE of 2.06555691 with a standard deviation of just $5.3799 \times 10^{-15}$. In contrast, competing algorithms like KOA and SWO show significant amounts of variability and much higher error rates; mean SSEs exceed 38.01, and standard deviations can reach 19.56.

**Table 6:** Statistical comparison of final SSE values of various algorithms for parameter extraction of different PEMFC stack.

Algorithms	Min	Mean	Max	std
	NedStack PS6			
THRO	2.06555691	2.06555691	2.06555691	$5.37987310 \times 10^{-15}$
FLA	2.06555691	4.60736946	2.44869645	$8.80089420 \times 10^{-01}$
ECO	2.06555691	5.64933571	2.21340425	$6.67288227 \times 10^{-01}$
KOA	3.89200357	$1.24771974 \times 10^{+01}$	$2.63386374 \times 10^{+01}$	6.68209841
FATA	2.16319829	3.62741805	2.78018368	$3.92901067 \times 10^{-01}$
SWO	3.49721451	8.33867609	$2.73542209 \times 10^{+01}$	$1.95692243 \times 10^{+01}$
	Horizon 500W			
THRO	$1.12418625\times 10^{-02}$	$1.12418625\times 10^{-02}$	$1.12418625\times 10^{-02}$	$2.86393688\times 10^{-13}$
FLA	$1.12418625\times 10^{-02}$	$3.47876483\times 10^{-02}$	$1.55339321\times 10^{-02}$	$8.33249584\times 10^{-03}$
ECO	$1.12558327\times 10^{-02}$	$1.36218225\times 10^{-01}$	$2.75290431\times 10^{-02}$	$2.51309829\times 10^{-02}$
KOA	$4.63095269 \times 10^{-01}$	1.75909627	5.49141777	1.09016815
FATA	$1.64944372\times 10^{-02}$	$8.42563547\times 10^{-02}$	$4.66745241\times 10^{-02}$	$1.92987683\times 10^{-02}$
SWO	1.05510745	$1.73339305\times 10^{+01}$	5.21906175	3.83001748
	BCS 500W			
THRO	$1.16977807\times 10^{-02}$	$1.16977807\times 10^{-02}$	$1.16977807\times 10^{-02}$	$3.92570829\times 10^{-16}$
FLA	$1.16977807\times 10^{-02}$	$2.53266592\times 10^{-02}$	$1.31241259\times 10^{-02}$	$4.13816669\times 10^{-03}$
ECO	$1.16977814\times 10^{-02}$	$2.72129354\times 10^{-02}$	$1.62396537\times 10^{-02}$	$5.43607094\times 10^{-03}$
KOA	$4.34256780\times 10^{-02}$	1.90101625	5.10066656	1.53955933
FATA	$1.20590785\times 10^{-02}$	$3.45329741\times 10^{-02}$	$1.74195648\times 10^{-02}$	$5.68964217\times 10^{-03}$
SWO	1.42626428	2.62717468	$1.16377128\times 10^{+01}$	7.00838915
	250W			
THRO	5.25142358	5.25142358	5.25142358	$2.84865341\times 10^{-12}$
FLA	5.25142358	5.79748891	5.31352615	$1.52705054\times 10^{-01}$
ECO	5.25150372	6.31603336	5.34710907	$2.19232103\times 10^{-01}$
KOA	6.24838811	8.40620024	$1.44406959\times 10^{+01}$	1.87107894
FATA	5.25183089	5.54040050	5.31601113	$8.07140527\times 10^{-02}$
SWO	7.12058612	$3.80103211\times 10^{+01}$	$1.35050427\times 10^{+01}$	6.37480789
	Avista SR-12			
THRO	1.05636978	1.05636978	1.05636978	$2.48127031\times 10^{-14}$
FLA	1.05636978	1.05679497	1.05955873	$1.10256711\times 10^{-3}$
ECO	1.05636978	1.05908013	1.09355484	$7.23606032\times 10^{-3}$
KOA	1.57817239	5.29699829	2.15372451	4.27343126
FATA	1.05884795	1.06981470	1.08979440	$8.23824276\times 10^{-3}$
SWO	2.34383753	11.0044695	20.2458283	4.99185149
	Ballard Mark V			
THRO	0.813911703	0.813911703	0.813911703	$1.77084503\times 10^{-15}$
FLA	0.813911703	0.813911707	0.813911819	$2.10171793\times 10^{-8}$
ECO	0.813911703	0.857538875	1.49066360	$1.39571390\times 10^{-1}$
KOA	1.03950265	4.59578417	14.9900611	3.61425640
FATA	0.814121754	0.838223222	0.924386811	$3.41010124\times 10^{-2}$
SWO	3.76396140	22.5871174	56.5724159	14.6218619

**Fig. 4:**
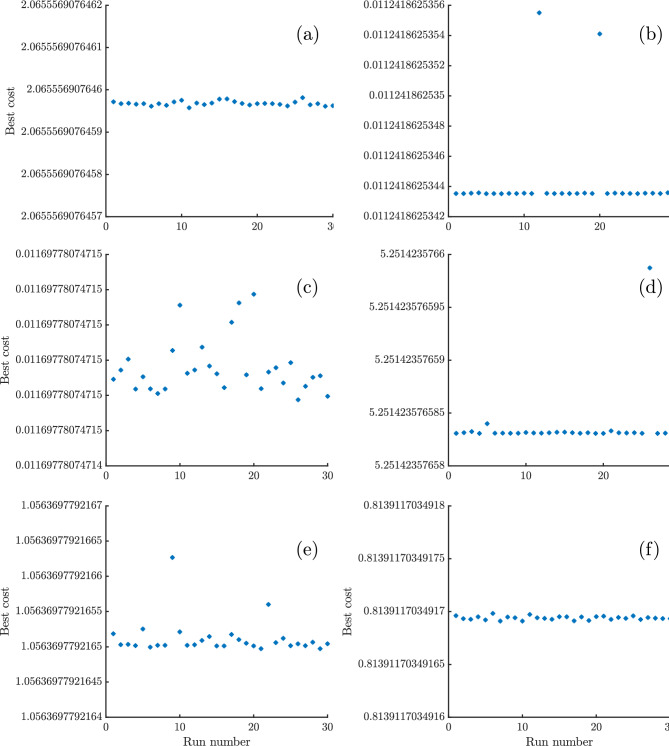
Objective function values obtained from each run of the THRO for: (**a**) NedStack PS6, (**b**) Horizon 500W, (**c**) BCS 500W, (**d**) 250W, (**e**) Avista SR-12, and (**f**) Ballard Mark V PEMFC stacks.

A similar pattern holds for the Horizon 500W, BCS 500W stacks, Avista SR-12, and Ballard Mark V where THRO consistently reports SSEs of 0.0112, 0.0117, 1.056, and 0.814 respectively, with negligible fluctuations. Finally, even for the more challenging 250W stack, THRO consistently yields an SSE of 5.25142358 with effectively zero spread. In contrast, the alternative algorithms (e.g., ECO with mean $SSE \simeq 6.32$ and $std \simeq 0.22$, or SWO with mean $SSE \simeq 38.01$ and $std \simeq 6.37$) struggle both in accuracy and stability. Ultimately, THRO comes out on top in this comparison study because it has the lowest errors with consistently lowest SSE with negligible deviation. THRO makes it an excellent choice for applications that need both accuracy and dependability. As shown in Fig. 4, the best cost values obtained from each run of the THRO demonstrate the algorithm’s superior performance in terms of accuracy, consistency, and reliability. The narrow dispersion of objective function values across independent runs highlights the robustness of the optimization process and its ability to avoid premature convergence. Moreover, the stability of the results across different PEMFC stack types confirms the generalizability of the THRO, indicating that the algorithm can effectively balance exploration and exploitation in diverse parameter landscapes.

**Fig. 5:**
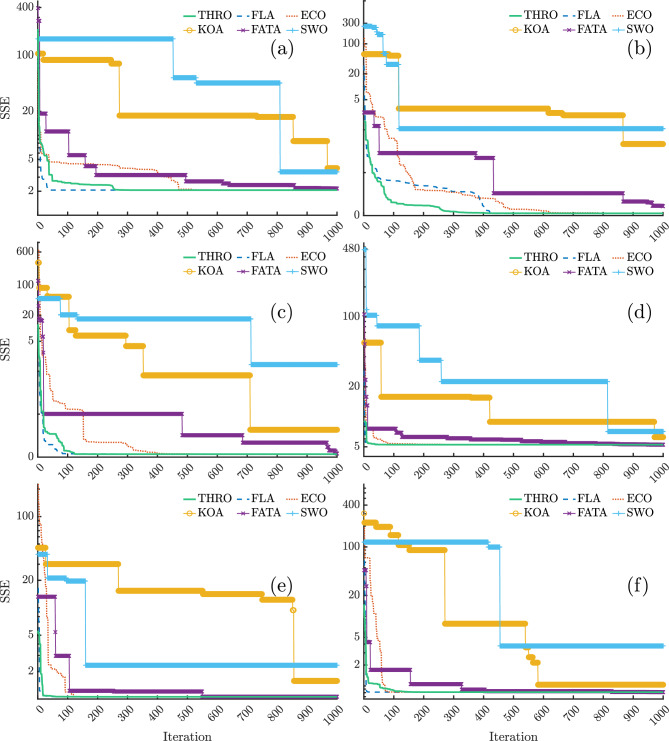
The convergence curves of different algorithms during the parameter extraction process of (**a**) NedStack PS6, (**b**) Horizon 500W, (**c**) BCS 500W, (**d**) 250W, (**e**) Avista SR-12, and (**f**) Ballard Mark V PEMFC stacks.

Under identical simulation conditions, the convergence curves of the best run of the proposed algorithms are illustrated in Fig. 5, showing the variation in SSE values during the parameter extraction process for the six aforementioned PEMFC stacks. Note that the y-axis is presented on a logarithmic scale. As shown in Fig. 5, THRO consistently demonstrates a superior convergence profile across all six PEMFC stacks.

In the NedStack PS6 case (Fig. 5a), THRO and FLA rapidly reduce the SSE from approximately 20 to below 5 in fewer than 50 iterations-outperforming all other algorithms. The curve then stabilizes around $SSE \simeq 2$ by iteration 60 for FLA and iteration 260 for THRO, with minimal changes observed up to iteration 1000.

In the Horizon 500W scenario (Fig. 5b), THRO again shows the steepest initial decline, reaching $SSE \simeq 10^{-2}$ within 260–300 iterations, followed by a stable plateau. In contrast, FLA and ECO converge more slowly, stabilizing around iteration 400 for FLA and iteration 600 for ECO.

For the BCS 500W case (Fig. 5c), THRO reduces the SSE to around $10^{-2}$ within approximately 120 iterations and maintains this value with no further variation. However, FLA achieves convergence even faster than THRO in this instance.

In the 250W stack extraction case (Fig. 5d), THRO decreases the SSE from around 20 to around 5 within just 20 iterations, then gradually stabilizes near $SSE \simeq 5$ by iteration 40, with negligible changes thereafter. Similar to the previous case, FLA demonstrates a faster convergence rate.

In the Avista SR-12 scenario (Fig. 5e), THRO and FLA converge to the optimal value in fewer than 50 iterations and outperform the other algorithms. In contrast, the case of the Ballard Mark V stack (Fig. 5f) shows that FLA reduces the SSE to the optimal value in fewer than 20 iterations, whereas THRO reaches the optimal value at around iteration 120.

In all six cases, THRO and FLA not only show the fastest initial convergence but also get the lowest and most stable final SSE values. This shows that they are effective and reliable for accurately identifying PEMFC parameters.

In contrast, ECO converges more slowly than THRO and FLA, while other competing methods, such as KOA, FATA, and SWO, display prolonged stagnation or recurring oscillations throughout the simulation.

The curves of Fig. 5, highlight the exploration-exploitation balance of the algorithms. THRO shows a well-managed dynamic, with rapid early exploration followed by stable exploitation near the optimum. FLA behaves similarly but sometimes converges faster due to stronger exploitation, which may risk local stagnation. ECO’s slower decline reflects broader exploration at the cost of delayed refinement, while KOA, FATA, and SWO suffer from imbalance, leading to oscillations or stagnation.

Table 7, showcases the optimal parameters obtained for various fuel cell models when subjected to different algorithms, alongside with the SSE values and elapsed time.

For the NedStack PS6 PEMFC model, various optimization algorithms have been employed for parameter extraction. Most algorithms produced relatively consistent SSE around 2.06, except FATA, KOA, and SWO, which resulted in higher SSE values of 2.16, 3.89, and 3.49, respectively. The THRO, FLA, and ECO algorithms yielded nearly identical results, particularly in terms of SSE. In contrast, KOA and FATA generated distinct parameter sets characterized by slightly higher values of $\lambda$ and $\beta$. Established methods from the literature, such as Dandelion Optimization (DO)^85^, Improved Fluid Search Optimization Algorithm (IFSOA)^48^, Improved Artificial Ecosystem Optimizer (IAEO)^95^ and Genetic Algorithm (GA)^96^, demonstrated comparable performance to the proposed algorithms, underscoring their continued relevance and competitiveness. In summary, this case study highlights the robustness of the THRO, FLA, and ECO algorithms in accurately extracting parameters for the NedStack PS6 PEMFC model while also revealing the limitations of specific other methods.

**Table 7:** The optimal parameter values, SSE values and elapsed time obtained from various algorithms for parameter extraction of different PEMFC stack.

Algorithms	$\xi _1$	$\xi _2 \times 10^{-3}$	$\xi _3 \times 10^{-5}$	$\xi _4 \times 10^{-5}$	$\lambda$	$\beta \times 10^{-2}(V)$	$R_c \times 10^{-4} (\Omega )$	SSE	t(s)
	NedStack PS6								
THRO	$-0.9146023053$	3.1103142926	7.4078557895	$-9.54$	12.5743308	1.36	1.00	2.06555691	5.7482
FLA	$-1.19969$	4.2686233306	9.7464882196	$-9.54$	12.5743308	1.36	1.00	2.06555691	5.2450
ECO	$-0.865900221$	3.3029610113	9.80	$-9.54$	12.57433079	1.36	1.00	2.06555691	2.0313
KOA	$-0.8642408028$	2.9791493620	7.4523003053	$-9.9892838702$	14.40717604	1.1984778415	1.2800677138	3.89200357	0.0502
FATA	$-1.117295336$	3.7927011832	8.0515827745	$-9.5408186921$	13.01080591	5.5567475188	1.0391432259	2.16319829	1.8432
SWO	$-0.9975894886$	3.2091662735	6.3146381704	$-9.7894330587$	13.73617894	7.3970813414	1.1518260327	3.49721451	0.0531
DO^85^	$-1.10823$	3.48488	5.23328	$-9.54$	23.0714	8.35662	1.27527	2.077565321	–
IFSOA^48^	$-0.92$	3.46	7.59	$-9.62$	13.15	4	0.10	2.15	1.28
GA^96^	$-1.1997$	3.4172	3.6000	$-9.5400$	13.0000	3.59	1.376	2.4089	–
IAEO^95^	$-0.9822$	3.5957	9.48	$-9.5400$	13.4650	1.36	1.00	2.1459	–
	Horizon 500W								
THRO	$-0.8532$	2.7832660365	9.80	$-15.516031380$	10	4.7746426902	8.00	$1.12418625\times 10^{-02}$	4.7326
FLA	$-0.8532$	2.7832660383	9.80	$-15.516031487$	10	4.7746426629	8.00	$1.12418625\times 10^{-02}$	4.6698
ECO	$-0.8532024799$	2.7638787560	9.6599509026	$-15.511432027$	10	4.7774265691	8.00	$1.12558327\times 10^{-02}$	1.7300
KOA	$-1.022812646$	2.6626697989	4.8833310813	$-14.873823065$	15.4097129	6.7256395062	3.3361596637	$4.63095269\times 10^{-01}$	0.0429
FATA	$-0.8532$	2.4195604080	7.1589772793	$-15.766769139$	10.16854404	4.9447646221	6.0350090777	$1.64944372\times 10^{-02}$	1.6522
SWO	$-1.143109158$	3.0417123430	4.1688715072	$-17.617847619$	12.46369824	6.0708286894	7.2398291767	1.05510745	0.0941
SMS^91^	$-0.8532$	2.78326602	9.80	$-15.51603042$	10.00	4.77464274	8.00	$1.124186\times 10^{-02}$	–
MS-TSO^97^	$-0.8532$	2.7833	9.8	$-15.516$	10.00	4.77	8.00	$1.12\times 10^{-02}$	–
PSO^97^	$-0.8532$	1.9302	3.6	$-15.57$	10.00	4.86	7.9996	$1.1787\times 10^{-02}$	–
	BCS 500W								
THRO	$-1.030312131$	3.0547161500	5.8105674797	$-19.301733594$	20.87724357	1.6126132910	1.00	$1.16977807 \times 10^{-02}$	4.8067
FLA	$-0.9904907362$	2.5927917531	3.6044730968	$-19.301733555$	20.87724341	1.6126132902	1.00	$1.16977807 \times 10^{-02}$	4.4986
ECO	$-1.16964463$	4.0912655890	9.7939124677	$-19.301659780$	20.87639785	1.6125537940	1.00	$1.16977814\times 10^{-02}$	1.6838
KOA	$-0.8532$	3.0404275774	9.1196181707	$-18.866718482$	18.51753069	1.36	3.4242083142	$4.34256780\times 10^{-02}$	0.2424
FATA	$-0.9613014791$	2.6634620678	4.6266112970	$-19.203413315$	23.28717609	1.6043063029	3.5419888664	$1.20590785\times 10^{-02}$	1.4347
SWO	$-1.043850523$	3.6949497714	9.6838436269	$-18.522947032$	21.30767385	2.2229642673	4.5880502038	1.42626428	0.0598
IAEO^95^	$-1.0750$	3.2100	5.95	$-19.0$	20.8772	1.61	1.00	$1.16\times 10^{-02}$	–
HHO^98^	$-1.09311$	3.28041	5.67397	$-18.9666$	20.0436	1.5148	2.25793	$1.4879\times 10^{-02}$	–
AEO^92^	$-0.85596$	2.73328	6.634280	$-19.2816$	20.702572	1.004526	01.6023	$1.157\times 10^{-02}$	–
CHHO4^99^	$-1.165963$	3.9095335	8.1088029	$-18.046841$	19.3423812	1.36016893	6.49013988	$5.9288512\times 10^{-02}$	–
THRO	$-1.19969$	3.5505839580	6.4098423913	$-12.138896719$	13.22968016	3.3344334275	1.00	5.25142358	7.4761
FLA	$-1.19969$	3.5505839824	6.4098425654	$-12.138896577$	13.22968031	3.3344335270	1.00	5.25142358	5.6568
ECO	$-0.8620527607$	2.8084611916	7.9276649336	$-12.146242921$	13.24541937	3.3371638164	1.00	5.25150372	2.8980
KOA	$-0.9927958058$	3.0080639856	6.7027637759	$-13.620879251$	15.44397611	2.7855212279	6.9214102919	6.24838811	0.1020
FATA	$-0.9592906149$	3.0256308986	7.5122416886	$-12.157382009$	13.2912209	3.3475416624	1.00	5.25183089	5.7078
SWO	$-0.857111759$	2.6039743590	6.5194390634	$-13.585744512$	13.67816979	2.3647670585	5.8565680690	7.12058612	0.1056
SMS^91^	$-1.19969$	3.55273318	6.43872061	$-12.13901908$	13.22868322	3.33438128	1.00	5.25133875	–
JAYA-NM^100^	$-1.19966$	3.55	6	$-12$	13.22870	3.334	1.00	5.2513	–
JAYA^100^	$-1.19969$	3.48	6	$-14$	15.21097	3.337	2.2	5.6918	–
	Avista SR-12								
THRO	$-1.071727171$	3.0727521947	4.3042416579	$-9.54$	24	17.537618953	6.8288101977	1.05636978	4.9116691
FLA	$-0.8663266858$	3.2865944138	9.7999981070	$-9.54$	24	17.537618997	6.82881011	1.05636978	4.5638895
ECO	$-1.109670769$	3.9627032578	9.3002042837	$-9.54$	24	17.537619028	6.8288097075	1.05636978	2.9478104
KOA	$-0.9324877531$	2.5366870202	3.7061325229	$-9.54$	11.31141309	17.879373295	1.7722087827	1.57817239	0.0990283
FATA	$-0.8968210656$	3.3808185416	9.80	$-9.54$	17.91203859	17.455780345	6.1352993339	1.05884795	1.5280259
SWO	$-1.01844911$	3.0283064187	5.1603138262	$-0.10742499885$	15.55763196	16.563791859	2.7480305359	2.34383753	0.0581734
MAEO^92^	$-0.86068$	2.77134	6.19649	$-9.54009$	22.98870	17.5366	6.70732	1.05663	–
DEA^87^	$-1.1021$	3.7687	8.2004	$-9.54$	24	17.69	8.00	1.05374	–
	Ballard Mark V								
THRO	$-1.08604672$	3.6960066540	6.8228474681	$-16.725137922$	24	1.5884374009	1.00	0.813911703	5.1397911
FLA	$-1.199260732$	4.4318028525	9.7231857179	$-16.725137909$	24	1.5884374024	1.00	0.813911703	4.4636364
ECO	$-1.199689993$	4.3852233888	9.3812656073	$-16.725137889$	24	1.5884374177	1.00	0.813911703	1.5905329
KOA	$-1.146636791$	3.9836888545	7.7752735956	$-15.428948621$	24	1.36	2.7954066440	1.03950265	0.0815583
FATA	$-0.8539724264$	2.6096639142	3.8861953182	$-16.764890669$	24	1.5795669968	1.0037224296	0.814121754	1.367805
SWO	$-1.15759913$	4.2791070633	9.2573526611	$-15.033490756$	23.26606413	1.9807992390	5.2902055840	3.76396140	0.0762793
ARO^101^	$-1.158859$	3.5208	4.0526	$-16.7251$	23.99	1.5884	1.00	0.8139117035	–
NNA^102^	$-0.97997$	3.6946	9.0871	$-16.2820$	23.00	1.36	1.00	0.85361	–

In the Horizon 500W case, the algorithms such as THRO, FLA, and ECO again showed near-identical results, with very low SSE values around $1.12 \times 10^{-2}$, indicating highly accurate parameter estimation. The SWO algorithms deviated significantly with much higher SSE of 1.05. Previously used methods from the literature, like Shuffled Multi-Simplexes search (SMS) algorithm^91^, Multi-Strategy Tuna Swarm Optimization (MS-TSO)^97^, and PSO^97^ maintained competitive performance, closely matching the new methods in terms of SSE. On the other hand, FATA shows an SSE value of $1.64 \times 10^{-2}$.

For the BCS 500W stack, THRO, FLA, and ECO once again achieved identical SSEs around $1.17 \times 10^{-2}$, demonstrating strong consistency. SWO again produced significantly higher SSE of 1.42. The literature methods, including IAEO^95^, Harris Hawks’ Optimization (HHO), and Artificial Ecosystem Optimizer (AEO)^92^, achieved similar SSE values, showcasing their reliability. However, CHHO4 (HHO based on the fourth chaotic function^99^) resulted in a notably worse performance with an SSE of $5.92 \times 10^{-2}$, possibly due to overfitting or convergence issues. This case emphasizes the robustness of specific modern algorithms and reveals the limitations of others when faced with increased model complexity.

In the 250W PEMFC case, the THRO, FLA, and ECO algorithms continued their trend of yielding consistent parameter values and SSEs near 5.25, demonstrating high reliability. KOA and SWO again introduced more variation in parameters, with significantly higher SSE values, reinforcing the pattern observed in previous stacks. The SMS^91^, JAYA-NM (Nelder-Mead), and JAYA methods^100^ from the literature performed on par with or slightly below the proposed methods, with JAYA showing a slightly higher SSE (5.69). This case further confirms the consistent accuracy of specific algorithms across different PEMFC stack types.

For the Avista SR-12 stack, the THRO, FLA, and ECO algorithms achieved nearly identical SSE values of approximately 1.056, confirming their reliable convergence and stable parameter extraction behavior. In contrast, KOA and SWO exhibited higher SSE values, along with noticeably different parameter sets, indicating less effective optimization for this stack. The literature methods, such as Modified Artificial Ecosystem Optimization (MAEO)^92^ and Differential Evolution Ameliorated (DEA)^87^, produced comparable SSE values close to 1.05, demonstrating strong agreement with the best-performing algorithms. Overall, this case highlights the robustness of THRO, FLA, and ECO for medium-sized stacks while also confirming the competitive performance of modern evolutionary approaches.

In the Ballard Mark V scenario, THRO, FLA, and ECO again showed highly consistent behavior, all achieving an SSE of approximately 0.814, reflecting precise model fitting. Other algorithms such as KOA and SWO produced higher SSE values, confirming their limited reliability for this stack. Comparative methods from the literature, including the Artificial Rabbits Optimization (ARO)^101^ and the Neural Network Algorithm (NNA)^102^, delivered similar or slightly higher SSEs, validating the challenging nature of this dataset. This case reinforces the effectiveness of the proposed algorithms in handling complex, large-scale PEMFC stacks with high accuracy.

From a practical deployment perspective, the computational efficiency of the proposed THRO algorithm was evaluated in comparison with state-of-the-art metaheuristic approaches. The results indicate that THRO achieves a competitive trade-off between solution accuracy and computational cost, reinforcing its suitability for PEMFC parameter estimation tasks. Although THRO may exhibit a higher computational time in certain cases, this behavior stems from its balanced exploration-exploitation mechanism, which enhances convergence stability and reduces the risk of premature stagnation. Importantly, the associated memory requirements remain modest, as THRO relies on a population-based structure with simple update rules. While computational overhead may represent a limitation for direct deployment in highly constrained embedded or real-time PEMFC control systems, the algorithm is well-suited for offline parameter identification. Moreover, implementation feasibility in real-time environments can be enhanced through population size reduction, iteration truncation, or hybrid integration with lightweight local search techniques.

**Fig. 6:**
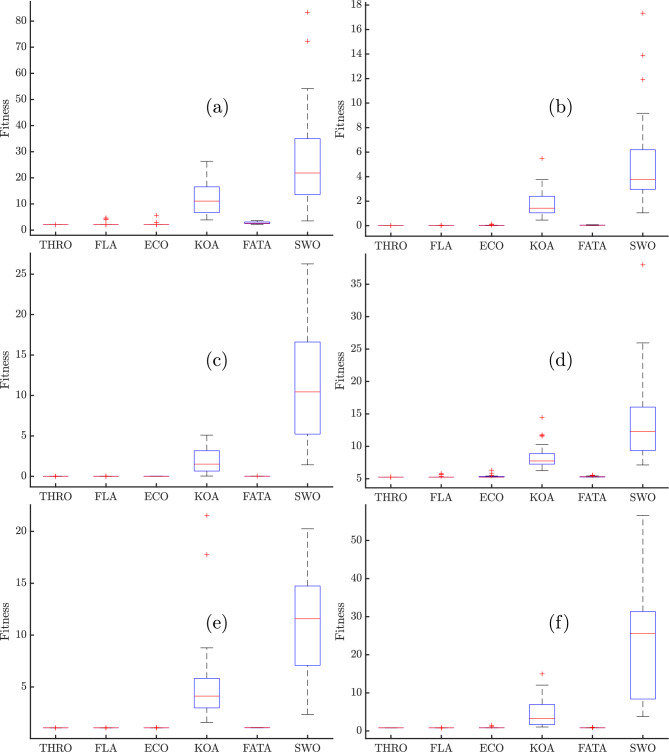
Box plot of different algorithms for (**a**) NedStack PS6, (**b**) Horizon 500W, (**c**) BCS 500W, (**d**) 250W PEMFC stacks, (**e**) Avista SR-12, and (**f**) Ballard Mark V stacks.

The box plots in Fig. 6 illustrate the fitness values of different algorithms across six PEMFC stacks. THRO and FLA consistently demonstrate the lowest and most stable fitness values, indicating superior and reliable optimization performance. In contrast, algorithms like SWO and KOA show higher fitness values with greater variability, suggesting less consistent outcomes. Overall, these results highlight the effectiveness of THRO and FLA in achieving robust solutions compared to the other methods.

The results in Table 8 clearly demonstrate the superior performance of the THRO algorithm across all PEMFC stack models. THRO consistently achieves the lowest values in all three performance metrics (MAE, RE, and RMSE) indicating exceptional accuracy and stability. The extremely small error magnitudes (on the order of $10^{-14}$ to $10^{-16}$) reflect minimal deviation from the best-obtained solution ($\text {SSE}_{\min }$), highlighting THRO’s robustness and precision in model fitting. These findings confirm THRO’s effectiveness as a highly reliable optimization method for PEMFC parameter estimation.

**Table 8:** Quantitative validation metrics across various algorithms and PEMFC stack models.

Algorithms	Metric	NedStack PS6	Horizon 500W	BCS 500W	250W	Avista SR-12	Ballard Mark V
THRO	MAE	$1.036208 \times 10^{-14}$	$7.705300 \times 10^{-14}$	$4.596439 \times 10^{-16}$	$6.077213 \times 10^{-13}$	$1.472156 \times 10^{-14}$	$2.923587 \times 10^{-15}$
	RE	$1.504981 \times 10^{-13}$	$2.056234 \times 10^{-10}$	$1.178798 \times 10^{-12}$	$3.471752 \times 10^{-12}$	$4.180797 \times 10^{-13}$	$1.077606 \times 10^{-13}$
	RMSE	$1.163028 \times 10^{-14}$	$2.919323 \times 10^{-13}$	$6.002061 \times 10^{-16}$	$2.865948 \times 10^{-12}$	$2.849295 \times 10^{-14}$	$3.401947 \times 10^{-15}$
FLA	MAE	0.383139	0.004292	0.001426	0.062102	$4.251947 \times 10^{-4}$	$3.842912\times 10^{-9}$
	RE	5.564691	11.453803	3.657989	0.354775	0.012075	$1.416460\times 10^{-7}$
	RMSE	0.946327	0.009249	0.004311	0.162475	0.001164	$2.101822 \times 10^{-8}$
ECO	MAE	0.147847	0.016273	0.004542	0.095605	0.002710	0.043627
	RE	2.147324	43.372741	11.648035	0.546159	0.076972	1.608055
	RMSE	0.672525	0.029586	0.007014	0.235798	0.007613	0.143993
KOA	MAE	8.585193	1.296001	1.857590	2.157812	3.718826	3.556282
	RE	66.175637	83.956871	1283.28951	10.360170	70.692389	1.026341
	RMSE	10.810534	1.681805	2.396221	2.835558	5.610984	5.027381
FATA	MAE	0.616985	0.030180	0.005360	0.064180	0.010967	0.024101
	RE	8.556571	54.891391	13.335561	0.366616	0.310717	0.888128
	RMSE	0.727939	0.035649	0.007748	0.102062	0.013634	0.041292
SWO	MAE	23.857006	4.163954	10.211448	6.384457	8.660631	18.823156
	RE	204.651499	118.394225	214.787301	26.898586	110.8519	1.500267
	RMSE	30.648754	5.614141	12.318845	8.946779	9.954622	23.685088

To statistically validate the superiority of THRO, the Wilcoxon rank-sum test was conducted to compare its performance against each competing algorithm over 30 independent runs. For all PEMFC stacks, the resulting p-values were below 0.05 in most cases, indicating that the improvement in SSE achieved by THRO is statistically significant at the 5% significance level.

Table 9 presents the p-values from the Wilcoxon rank-sum test for each benchmark problem, comparing THRO to alternative algorithms. The symbols “+”, “$=$”, and “−” indicate whether THRO performed significantly better, statistically equivalent, or significantly worse than the competitor, respectively, based on a 0.05 significance threshold.

The results presented in Table 9 demonstrate the statistical superiority of the proposed THRO algorithm over five competing methods across six different fuel cell benchmarks. The extremely low p-values strongly reject the null hypothesis, indicating that the performance differences between THRO and its counterparts are statistically significant. Furthermore, the consistent presence of the “+” symbol across all comparisons confirms that THRO significantly outperforms all competitors on every benchmark considered. These findings highlight the robustness and effectiveness of THRO in optimizing fuel cell operation, regardless of the specific benchmark used.

**Table 9:** Wilcoxon rank-sum test *p*-values: THRO vs. competing algorithms.

THRO vs	NedStack PS6		Horizon 500W		BCS 500W		250W		Avista SR-12		Ballard Mark V	
	*p*-value		*p*-value		*p*-value		*p*-value		*p*-value		*p*-value	
FLA	$2.1006\times 10^{-10}$	+	0.0003	+	$1.2386\times 10^{-06}$	+	$3.3789\times 10^{-07}$	+	$7.438 \times 10^{-6}$	+	$5.019 \times 10^{-10}$	+
ECO	$1.4296\times 10^{-10}$	+	$3.0198\times 10^{-11}$	+	$3.0198\times 10^{-11}$	+	$3.0122\times 10^{-11}$	+	$1 \times 10^{-10}$	+	$7.344 \times 10^{-10}$	+
KOA	$2.9486\times 10^{-11}$	+	$3.0198\times 10^{-11}$	+	$3.0198\times 10^{-11}$	+	$3.0123\times 10^{-11}$	+	$3\times 10^{-11}$	+	$3.00 \times 10^{-11}$	+
FATA	$2.9487\times 10^{-11}$	+	$3.0199\times 10^{-11}$	+	$3.0199\times 10^{-11}$	+	$3.0123\times 10^{-11}$	+	$3\times 10^{-11}$	+	$3.00 \times 10^{-11}$	+
SWO	$2.9487\times 10^{-11}$	+	$3.0199\times 10^{-11}$	+	$3.0199\times 10^{-11}$	+	$3.0123\times 10^{-11}$	+	$3\times 10^{-11}$	+	$3.00 \times 10^{-11}$	+

**Fig. 7:**
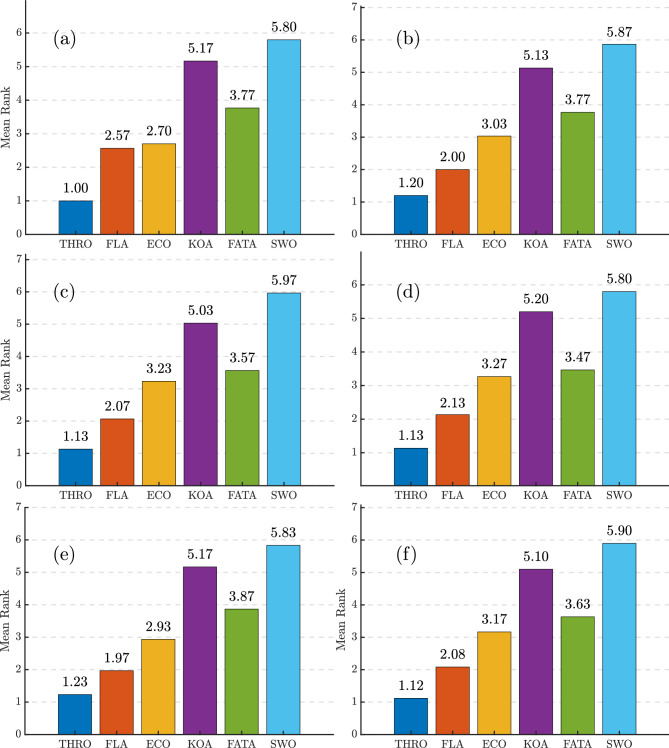
Mean rankings from Friedman test of (**a**) NedStack PS6, (**b**) Horizon 500W, (**c**) BCS 500W, (**d**) 250W, (**e**) Avista SR-12, and (**f**) Ballard Mark V PEMFC stacks.

Figure 7 presents the mean rankings derived from the Friedman test for six optimization algorithms across six different PEMFC stacks. In all cases, the THRO algorithm consistently achieves the best performance, obtaining the lowest mean rank, while SWO performs the worst, with the highest mean rank across all stacks. The results highlight the superior consistency and robustness of the THRO method in optimizing PEMFC operations, indicating its potential as a preferred approach for parameters extraction of PEMFC.

The statistical test of THRO provides additional evidence of its effectiveness beyond the numerical outcomes. In contrast to algorithms like FLA and ECO, which can suffer from oscillations or premature stagnation when exploration and exploitation are not well balanced, THRO follows a steadier search trajectory. Its iterations show a smooth and rapid reduction in SSE values, reflecting a more controlled search process. This balance between exploration and exploitation allows the method to avoid local optima with greater reliability and to converge accurately toward the global minimum. Taken together, these features highlight not only THRO’s efficiency but also its robustness, complementing the numerical results with stable and consistent convergence patterns.

**Fig. 8:**
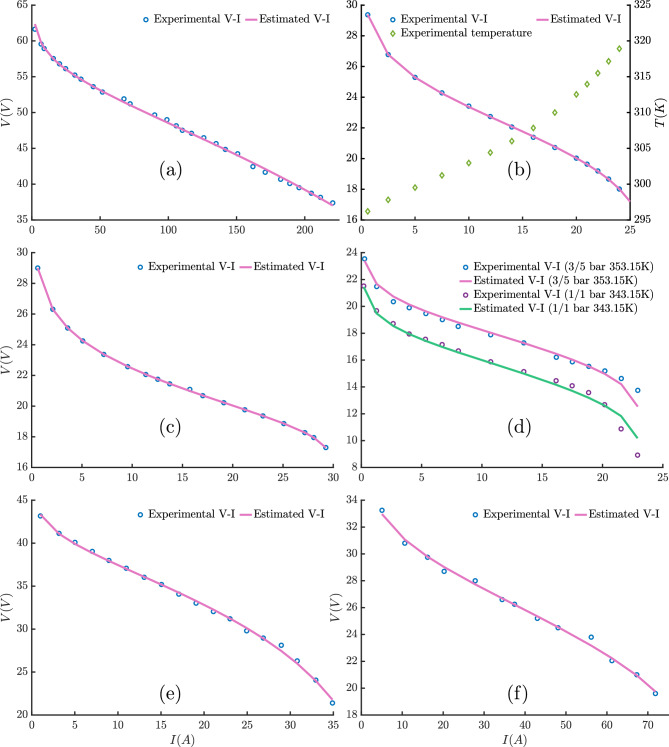
Estimated I-V polarization curves using THRO and experimental curves of (**a**) NedStack PS6, (**b**) Horizon 500W, (**c**) BCS 500W, (**d**) 250W, (**e**) Avista SR-12, and (**f**) Ballard Mark V PEMFC stacks.

Figure 8 presents a detailed comparison between the experimental data and the values estimated by the THRO algorithm for the NedStack PS6, Horizon 500W, BCS 500W, 250W, Avista SR-12, and Ballard Mark V PEMFC stacks. The close alignment between the estimated and measured voltages demonstrates the high accuracy of the model and the effectiveness of the parameter estimation process.

Figure 8a shows the voltage-current (V–I) polarization curve for the NedStack PS6 stack. The estimated V–I curve closely follows the experimental data over the entire current range, indicating reliable model performance even at high current densities.

In Fig. 8b, the Horizon 500W stack results are shown. In addition to the V–I behavior, the temperature variation during operation is presented. The model accurately predicts the voltage under varying thermal conditions, further validating the thermal-electrochemical coupling in the model.

Figure 8c, e and f corresponds to the BCS 500W, Avista SR-12, and Ballard Mark V PEMFC stacks, respectively. Similar to the previous cases, the estimated curve exhibits a strong correlation with the experimental data, reflecting the model’s ability to generalize across different stack configurations.

Finally, Fig. 8d compares two sets of experimental and estimated data for the 250W PEMFC stack under two operating conditions: 3/5 bar at 353.15 K and 1/1 bar at 343.15 K. The THRO algorithm effectively captures the distinct polarization behaviors under varying pressure and temperature, as evidenced by the close match between experimental and simulated curves in both scenarios.

**Fig. 9:**
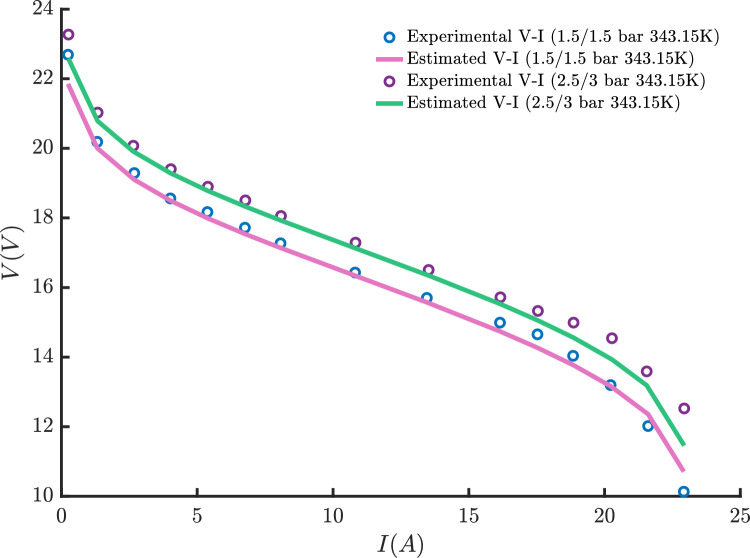
Model validation curves and experimental data of 250W PEMFC stacks.

Figure 9 presents the model validation results of 250W using the two independent experimental datasets at operating conditions of 2.5/3 bar and 1.5/1.5 bar, both at 343.15 K. These datasets were not used in the parameter estimation process and serve to evaluate the generalization capability of the THRO model. As shown, the estimated V–I curves align well with the experimental measurements under both conditions, demonstrating the model’s robustness and predictive accuracy. The close match shows that the model’s estimated parameters can be used to accurately predict the polarization changes with different pressure settings of the 250W PEMFC stacks. This validation reinforces the model’s potential for reliable performance forecasting under diverse operating scenarios.

**Fig. 10:**
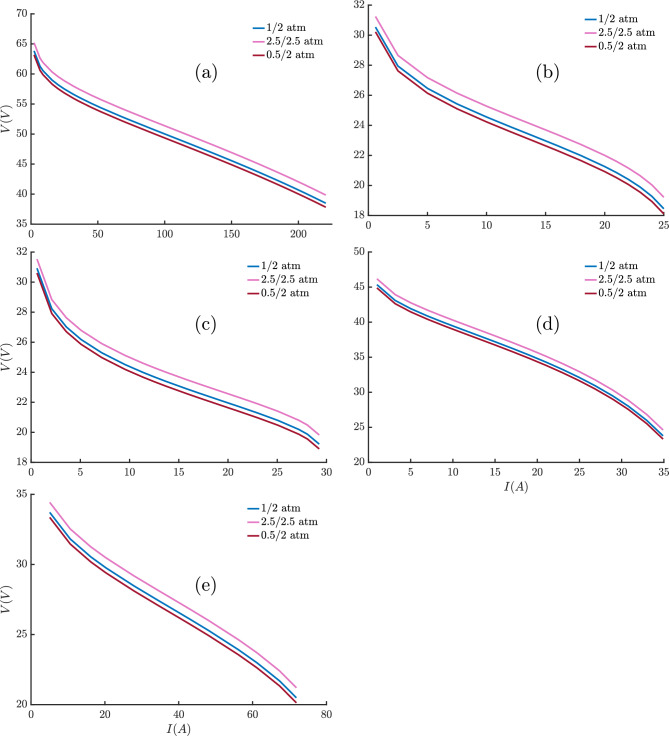
Variation of hydrogen and oxygen pressures, V–I curves for (**a**) NedStack PS6 (**b**) Horizon 500W (**c**) BCS 500W (**d**) Avista SR-12, and (**e**) Ballard Mark V PEMFC stacks.

Figure 10 illustrates the estimated polarization curves of the NedStack PS6, Horizon 500W, BCS 500W, Avista SR-12, and Ballard Mark V PEMFC stacks under varying $H_2$ and $O_2$ pressures, with the temperature set to their datasheet values, based on the optimal parameters obtained from the THRO.

**Fig. 11:**
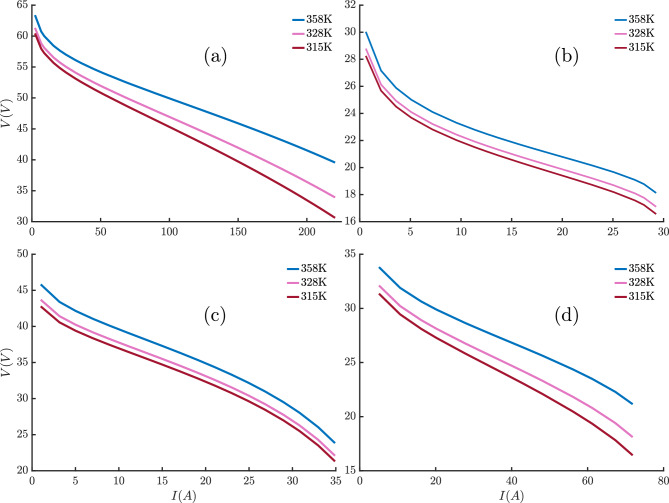
Temperature variation, V–I curves for (**a**) NedStack PS6 (**b**) BCS 500W (**c**) Avista SR-12, and (**d**) Ballard Mark V PEMFC stacks.

The stability and accuracy of the THRO are evaluated by analyzing the effects of varying cell temperature and reactant pressures. Figure 11 show the estimated polarization curves of the NedStack PS6, BCS 500W, Avista SR-12, and Ballard Mark V PEMFC stacks at different cell temperatures, with $H_2$ and $O_2$ pressures set to their datasheet values, based on the optimal parameters obtained from the THRO.

The results presented in Figs. 10 and 11 clearly demonstrate the robustness and validity of the parameters extracted using THRO across different PEMFC stacks. By accurately reproducing polarization behavior under variations in reactant pressures and cell temperatures, the proposed approach proves its capability to capture real-world operating conditions with high fidelity. This highlights the effectiveness of THRO in delivering reliable parameter estimation that ensures both practical relevance and predictive accuracy.

## Conclusion and future work

This study present an effective and robust parameter estimation framework for Proton Exchange Membrane Fuel Cell (PEMFC) models based on the recently proposed Tianji’s Horse Racing Optimization (THRO) algorithm. The proposed approach was systematically evaluated against five state-of-the-art metaheuristic algorithms, namely, Flood Algorithm (FLA), Educational Competition Optimizer (ECO), Kepler Optimization Algorithm (KOA), Fata Morgana Algorithm (FATA), and Spider Wasp Optimizer (SWO). These methods were used to extract the unknown parameters of six PEMFC systems: NedStack PS6, Horizon 500W, BCS 500W, 250W, Avista SR-12, and Ballard Mark V PEMFC stacks.

Applying THRO to the six PEMFC models yielded the following sum of squared errors (SSE): 2.06555691 for NedStack PS6, $1.12418625 \times 10^{-02}$ for Horizon 500W, $1.16977807 \times 10^{-02}$ for BCS 500W, 5.25142358 for 250W, 1.05636978 for Avista SR-12, and 0.813911703 for Ballard Mark V. The obtained results demonstrate that THRO consistently achieves superior parameter identification accuracy across all tested PEMFC models, as reflected by lower SSE values and reduced dispersion in statistical performance indicators. These results confirm the strong robustness and stability of THRO, particularly in avoiding premature convergence and maintaining reliable performance across different PEMFC configurations. Although FLA exhibited faster convergence in some cases, THRO provided improved solution accuracy and statistical consistency, highlighting a favorable trade-off between convergence reliability and optimization precision. However, this improved accuracy and robustness are achieved at the expense of slightly higher computational time compared to some competing algorithms, which represents the main limitation of the proposed THRO approach.

In addition, the effectiveness of the proposed method was further validated using multiple experimental datasets for the 250W PEMFC stack, where parameters extracted by THRO demonstrated strong generalization capability when applied to independent validation datasets. The robustness of the identified parameters under varying operating conditions, including changes in hydrogen and oxygen pressures as well as temperature variations, further confirms the practical applicability of the proposed approach in realistic PEMFC environments.

The main contributions of this work include the first application of the THRO algorithm to PEMFC parameter estimation, providing a novel optimization perspective for fuel cell modeling. In addition, a comprehensive comparative analysis is conducted against several recent state-of-the-art metaheuristic optimization algorithms to rigorously assess performance. The proposed approach is extensively validated using multiple commercial PEMFC stacks, demonstrating its generality and scalability across different system configurations. Furthermore, the robustness of the extracted parameters is evaluated under varying operating and environmental conditions, confirming the reliability and practical applicability of the THRO-based framework.

Future research could focus on the following directions:

- Developing a hybrid approach that combines the strengths of FLA and THRO to enhance the convergence speed of THRO.
- Reducing the computational time of THRO through algorithmic simplification or parallel processing techniques to improve its practicality in real-time applications.
- Applying the method to larger datasets from commercial PEMFC stacks and extracting model parameters under fault conditions in PEMFC cells.
- Extending THRO to more advanced PEMFC models, including dynamic and degradation-aware frameworks, and validating its scalability on larger and industrial stacks.
- Investigating hybridization with adaptive learning strategies to enhance performance in high-dimensional optimization problems.
- Exploring practical implementation in real-world PEMFC control systems, particularly for online monitoring, fault diagnosis, and hardware-in-the-loop validation, where the robustness and parameter-free nature of THRO are expected to offer significant benefits.
- Investigating the influence of relative humidity variations (e.g., 40%, 70%, 100% RH) to assess membrane hydration effects and further validate the robustness of the proposed method.

## Data Availability

The datasets used and/or analysed during the current study available from the corresponding author on reasonable request.
